# Downregulation of the Ubiquitin-E3 Ligase RNF123 Promotes Upregulation of the NF-κB1 Target SerpinE1 in Aggressive Glioblastoma Tumors

**DOI:** 10.3390/cancers12051081

**Published:** 2020-04-27

**Authors:** Xiaowen Wang, Matias A. Bustos, Xiaoqing Zhang, Romela Irene Ramos, Cong Tan, Yuuki Iida, Shu-Ching Chang, Matthew P. Salomon, Kevin Tran, Rebecca Gentry, Yelena Kravtsova-Ivantsiv, Daniel F. Kelly, Gordon B. Mills, Aaron Ciechanover, Ying Mao, Dave S.B. Hoon

**Affiliations:** 1 Department of Translational Molecular Medicine, John Wayne Cancer Institute (JWCI) at Providence Saint John’s Health Center, Santa Monica, CA 90404, USA; wangxw14@fudan.edu.cn (X.W.); Bustosm@jwci.org (M.A.B.); Xiaoqing.Zhang@providence.org (X.Z.); Romela.Ramos@providence.org (R.I.R.); yiida-tky@umin.ac.jp (Y.I.); msalomon@usc.edu (M.P.S.); Kevin.Tran@providence.org (K.T.); Rebecca.Gentry@providence.org (R.G.); maoying@fudan.edu.cn (Y.M.); 2 Department of Neurosurgery, Huashan Hospital, Fudan University, Shanghai 200040, China; 3 Department of Pathology, Cancer Hospital, Fudan University, Shanghai 200032, China; saratancong@163.com; 4 Medical Data Research Center, Providence Saint Joseph’s Health, Portland, OR 97225, USA; ShuChing.Chang@providence.org; 5 The David and Janet Polak Cancer and Vascular Biology Research Center, The Rappaport Faculty of Medicine and Research Institute, Technion-Israel Institute of Technology, Efron Street, Bat-Galim, Haifa 31096, Israel; yelenaiv@technion.ac.il (Y.K.-I.); aaroncie@technion.ac.il (A.C.); 6 Pacific Neuroscience Institute, JWCI, Santa Monica, CA 90404, USA; KellyD@JWCI.ORG; 7 Department of Cell Development and Cancer Biology, Knight Cancer Institute, Portland, OR 97239, USA; millsg@ohsu.edu; 8 The Collaborative Innovation Center for Brain Science, Fudan University, Shanghai 200032, China

**Keywords:** p50, miR-155, PAI-1, KPC1, NF-κB pathway

## Abstract

This study examined the role of the ubiquitin E3-ligase RNF123 in modulating downstream NF-κB1 targets in glioblastoma (GB) tumor progression. Our findings revealed an oncogenic pathway (miR-155-5p-RNF123-NF-κB1-p50-SerpinE1) that may represent a new therapeutic target pathway for GB patients with isocitrate dehydrogenase 1 and 2 (*IDH*) WT (wild type). Mechanistically, we demonstrated that *RNF123* is downregulated in *IDH* WT GB patients and leads to the reduction of p50 levels. RNA-sequencing, reverse-phase protein arrays, and in vitro functional assays on *IDH* WT GB cell lines with RNF123 overexpression showed that SerpinE1 was a downstream target that is negatively regulated by RNF123. *SERPINE1* knockdown reduced the proliferation and invasion of *IDH* WT GB cell lines. Both SerpinE1 and miR-155-5p overexpression negatively modulated RNF123 expression. In clinical translational analysis, RNF123, SerpinE1, and miR-155-5p were all associated with poor outcomes in GB patients. Multivariable analysis in *IDH* WT GB patients showed that concurrent low RNF123 and high SerpinE1 was an independent prognostic factor in predicting poor overall survival (*p* < 0.001, hazard ratio (HR) = 2.93, 95% confidence interval (CI) 1.7–5.05), and an increased risk of recurrence (*p* < 0.001, relative risk (RR) = 3.56, 95% CI 1.61–7.83).

## 1. Introduction

Glioblastoma (GB) is one of the most common, and most aggressive, intracranial malignancies in humans [1]. In the current standard of care, the median survival time in GB patients is around 12–15 months after diagnosis [2,3,4]. Although considerable basic and clinical research advancement has occurred over the past decade, there is still a need for: (a) improvement in staging and treatment, (b) understanding the molecular pathways that drive pathobiology in GB patients, (c) identifying targets for treatment, and (d) finding prognostic factors associated with overall survival (OS) and recurrence in particular subgroup GB populations [2,4,5]. According to the WHO (World Health Organization), GB patients are classified into three subgroups based on the isocitrate dehydrogenase 1 (*IDH1*) and 2 (*IDH2*) genes mutation status: *IDH* mutant, *IDH* WT and NOS (not otherwise specified) [6]. *IDH* WT GB has a very poor disease outcome [7]. Despite the efforts made to classify GB tumors, the majority of GB patients receive the same treatments [4].

The ubiquitin-proteasome system (UPS) plays critical functions to maintain cellular homeostasis. As such the UPS controls the turnover of vital proteins that function in diverse processes such as the cell cycle, DNA-damage repair, cell metabolism, and cell stress [8,9,10,11]. The UPS controls a myriad of cell signaling pathways involved in the cell’s inflammatory response, growth, migration, invasion, and homeostasis. The UPS system relies on three types of enzymes: ubiquitin-activating-enzymes (E1), ubiquitin-conjugating enzymes (E2), and ubiquitin-ligases (E3). Ubiquitination is catalyzed by a cascade of reversible enzymatic reactions that play a critical role. The genome has ~600 E3 ligases and they possess catalytic activity that mediates the Ubiquitin ligation and confers specificity in recognizing the substrates. Thus, it is important to understand the biological functions and the role E3-ligases play in GB progression by modulating specific oncogenic pathways [12].

The nuclear factor kappa-light-chain-enhancer of the activated B cells (NF-κB) pathway plays an important role in the pathobiology and therapeutic response of GB [13,14,15]. The NF-κB family of transcription factors (RelA, c-Rel, RelB, NF-κB1 (p105/p50), and NF-κB2 (p100/p52)) comprises important mediators of the signaling pathways involved in the immune or inflammatory responses, cell proliferation, differentiation, and progression of multiple tumor types [13,14,15]. NF-κB1 (p105) and NF-κB2 (p100) are normally processed and activated into p50 and p52, respectively, by the proteasome system [16]. NF-κB1, or p105, is ubiquitinated by the ubiquitin-E3 ligase KIP1 ubiquitination-promoting complex subunit 1 (KPC1, also called RNF123 for ring finger protein 123) [9]. Ubiquitinated NF-κB1 is processed in the proteasome, which results in the formation and accumulation of p50 in different tumor types [9]. However, studies have reported that RNF123 also modulates the expression of p27 (CDKN1B) in fibroblasts and astrocytes in the spinal cord [17,18]. P50 homodimerizes forming p50–p50 dimers or heterodimerizes with other protein partners such as p65 to form p50–p65 dimers. P50 homodimers lack transactivation domains and hence act as suppressive factors that negatively regulate NF-κB target genes in different types of human solid tumors [9,19]. The aberrant regulation of the processing and activation of NF-κB1 under basal and induced conditions is associated with GB tumorigenesis [19]. The mechanisms governing the NF-κB1 activation into p50 and the downstream genes that are activated in GB progression are still not understood well. Research in this area is crucial for identifying theranostic targets in GB that will improve OS.

The serine protease inhibitor family E member 1 (SerpinE1, also known as plasminogen activator inhibitor-1 (PAI-1)) is an NF-κB1-pathway target that functions as an endogenous inhibitor of the serine protease urokinase-type plasminogen activator (uPA) [20]. The role of SerpinE1 in cancer does not seem to only be associated with the plasminogen activation system itself [21]. Previous reports have shown that SerpinE1 expression promotes angiogenesis and tumor cell survival by preventing apoptosis [22,23,24,25]. Induction of uPAR/SerpinE1 expression by sphingosine-1-phosphate and interleukin-1 has been shown to promote the invasiveness of U373 glioblastoma cell lines [26]. Studies have shown a strong correlation between SerpinE1 expression and poor prognosis in different types of solid tumors [22,27,28,29,30,31,32,33] as well as GB [34,35,36]. Moreover, increased serum levels of SerpinE1 were associated with poor survival in patients with high-grade gliomas, suggesting SerpinE1 utility as a blood marker [37]. Understanding SerpinE1′s cancer-related functions and the pathways that control SerpinE1 expression would aid in the development of new theranostic strategies for GB tumors.

In the present study, we hypothesized that RNF123 aberrant expression in *IDH* WT GB affects NF-κB1 processing and downstream targets, promoting GB tumor progression. Here, we identified NF-κB1 targets by using a combination of in silico, RNA-sequencing, and RPPA analysis with in vitro cell line models and functional assays. Our proposed mechanistic model was to understand the role of RNF123 in controlling SerpinE1 expression, and the regulation of RNF123 by miR-155-5p and SerpinE1 in aggressive *IDH* WT GB tumors. Of clinical relevance, patients with low RNF123 and high SerpinE1 expression had a poor OS and an increased risk of recurrence in *IDH* WT GB.

## 2. Results

### 2.1. RNF123 Downregulation is Associated with a Poor Prognosis in GB

**Figure 1:**
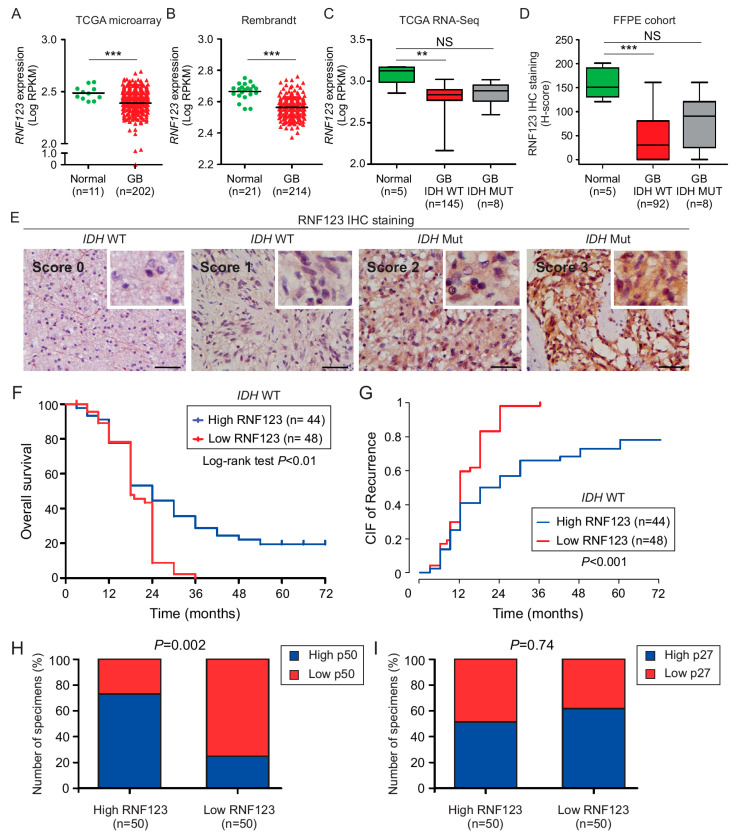
Analysis of RNF123 protein and mRNA expression in GB tumors. (**A**,**B**). Dot plot of *RNF123* mRNA expression in GB (*n* = 202; *n* = 214) tumors compared to normal brain tissue (*n* = 11; *n* = 21, respectively) using the microarray GB TCGA database (**A**) or GB Rembrandt dataset (**B**) (*t*-test *** *p* < 0.001). (**C**) Boxplot of *RNF123* expression in *IDH* WT (*n* = 145) or mutated (*n* = 8) GB patients using RNA-sequencing TCGA dataset and compared to normal brain tissue (*n* = 5) (Kruskal–Wallis test, Dunn’s post hoc test ** *p* < 0.01, NS = non-significant). (**D**) Boxplot of RNF123 expression in FFPE samples from normal brain tissue (*n* = 5), and from *IDH* WT (*n* = 92) or mutated (*n* = 8) GB patients as determined by H-scores (Kruskal–Wallis test, Dunn’s post hoc test *** *p* < 0.001, NS = non-significant). (**E**) Representative IHC images for RNF123 staining in FFPE samples showing the scores (0, 1, 2, or 3). Scale bar = 50 μm (**F**) Kaplan–Meier curves to compare the percentage of survival in GB patients with low (*n* = 48) versus high (*n* = 44) RNF123 expression (log-rank test, *p* < 0.01). (**G**) CIF of recurrence in GB patients with low (*n* = 48) versus high (*n* = 44) RNF123 expression (*p* < 0.001). (**H**,**I**) FFPE samples derived from GB patients were classified as high or low RNF123 expression based on the median of H-scores and analyzed by IHC for p50 (**H**) or p27 (**I**). The percentages of patients with high or low expression of p50 (**H**) or p27 (**I**) are shown (Fisher’s exact test, *p* = 0.002 (**H**) and *p* = 0.74 (**I**)).

The mRNA expression levels of *RNF123* in GB and normal brain tissues were compared using public databases. Both the GB TCGA (Figure 1A) and the GB Rembrandt dataset (Figure 1B) revealed that the expression of *RNF123* was significantly reduced in GB compared to normal brain tissue. According to the WHO 2016, GB tumors are stratified into the *IDH* WT and *IDH* mutated groups, since the former group of patients showed worse prognosis [6,38]. By using the RNA-sequencing data from the TCGA dataset for GB, brain tumors with *IDH* WT status showed a significant reduction in *RNF123* expression compared to normal brain tissue, but not in *IDH* mutated GB compared to normal brain tissue (Figure 1C). These results were also validated using a larger cohort of normal brain tissue from the GTEX dataset using the GEPIA database (Figure S2A). To confirm our in silico observations, an independent formalin-fixed paraffin-embedded (FFPE) cohort of well-annotated clinical specimens was stained for RNF123. The clinical features of the patients that were included in the tissue microarray (TMA1, containing 100 GB patients and 5 normal brain tissues) are described in the Materials and Methods section and Table S1A. Immunohistochemistry (IHC) analysis also revealed significantly reduced RNF123 protein levels in *IDH* WT GB, but not in *IDH* mutated GB compared to normal brain tissue (Figure 1D,E). Next, we assessed whether RNF123 expression is associated with OS in *IDH* WT GB tumors using the H-score values. To do that, GB patients with *IDH* mutation were excluded from the analysis. The results demonstrated that GB patients with *IDH* WT and low RNF123 expression have a poorer prognosis than those with *IDH* WT and high RNF123 expression (Figure 1F). To determine the prognostic relevance of RNF123 expression in GB tumors, univariable and multivariable analyses [39] were performed in patients with high or low RNF123 expression using the H-score values median cutoff and well-annotated clinical prognostic factors such as age at diagnosis, gender, *IDH* status, methyl guanine methyltransferase (*MGMT*) promoter methylation status and Karnofsky performance score (KPS), Table S1A). Importantly, in the multivariable analysis, the two independent factors associated with poor OS were low RNF123 expression (*p* < 0.02, HR = 2.15, 95% CI, 1.15–4.02) and KPS (*p* < 0.01, hazard ratio (HR) = 1.86, 95% CI), 1.14–3.05) and KPS < 85 (*p* < 0.01, HR = 2.10, 95% CI, 1.28–3.44; *p* < 0.003 Table S1B). Competing risks regression was used to evaluate the effect of RNF123 on progression-free survival (PFS), taking the competing risk of mortality (or death) into account. Cumulative incidence function (CIF) showed that low RNF123 expression had higher risks of recurrence than high RNF123 expression (*p* < 0.001, RR = 2.36, 95% CI, 1.54–3.61; Table S2A; Figure 1G). In summary, RNF123 expression is decreased in *IDH* WT GB patients and low RNF123 expression in *IDH* WT GB is negatively associated with prognosis and a higher probability of recurrence.

### 2.2. P50 Protein Levels but Not p27 Are Associated with RNF123 Expression in GB

RNF123 has been reported to be involved in the processing of p27 [17,40]. To determine if p50 and p27 levels were associated with RNF123 expression in GB, clinically annotated tumor samples with high (*n* = 50) and low (*n* = 50) RNF123 expression were immunostained for p50 and p27. GB with high RNF123 expression had significantly elevated p50 levels compared to tumors with low RNF123 expression (Figure 1H; Figure S1A), whereas there was no significant change in p27 levels (Figure 1I; Figure S1B). This indicated that RNF123 expression is associated with p50 but not with p27, reinforcing the hypothesis that RNF123 is a key processing factor for p105 into p50.

### 2.3. RNF123 Overexpression Suppresses Cell Proliferation and Invasion

**Figure 2:**
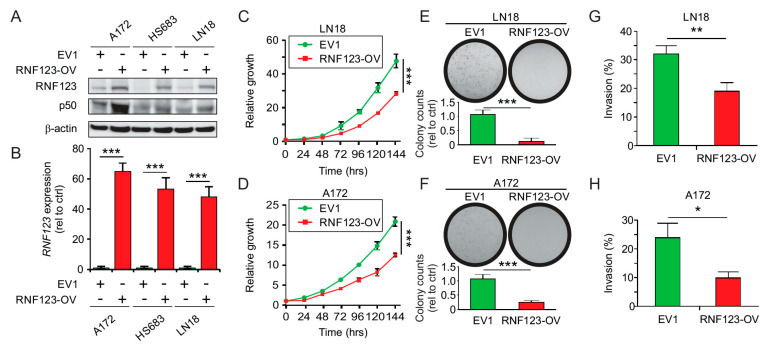

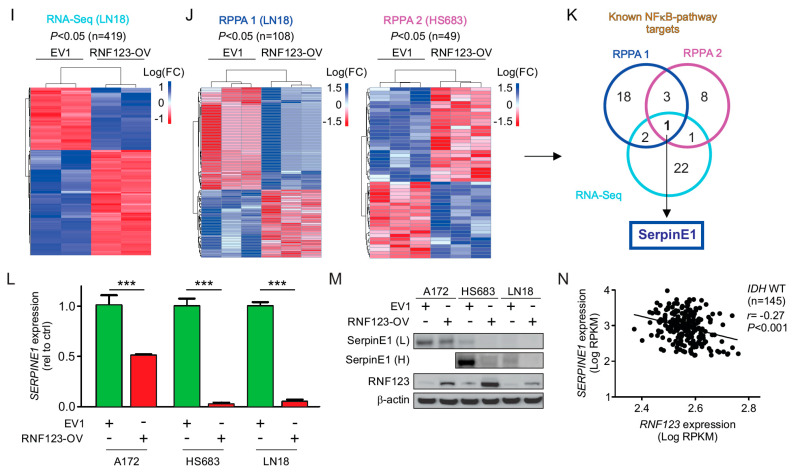
Analysis of cell proliferation and invasion in GB cells with RNF123-OV. (**A**) A172, HS683, and LN18 cell lines were stably transfected with empty vector (EV1) or a cDNA encoding Myc-*RNF123* (RNF123-OV). RNF123-OV and p50 were assessed by Western blot, and β-actin was used as a loading control. (**B**) Quantification of *RNF123* expression by RT-qPCR (*t*-test, *** *p* < 0.001). (**C**,**D**) Proliferation of LN18 (C) and A172 (D) cell lines with RNF123-OV or the empty vector (EV1) (two-way ANOVA, Bonferroni correction *** *p* < 0.001). (**E**,**F**) Colony-forming units LN18 (E) and A172 (F) cell lines stably expressing control empty vector 1 (EV1) or RNF123-OV (*t*-test, *** *p* < 0.001). (**G**,**H**) Percentage of invasion in LN18 (G) and A172 (H) cell lines stably expressing control (EV1) or RNF123-OV (*t*-test, * *p* < 0.05, ** *p* < 0.01). (**I**) LN18 cell lines expressing control vector (EV1) or RNF123-OV were analyzed by RNA-sequencing to determine differentially expressed (DE) genes in RNF123-OV cell lines. The image shows a heatmap of the most DE genes (adjusted *p* < 0.05). (**J**) LN18 (RPPA1) and HS683 (RPPA2) cell lines with RNF123-OV were analyzed by RPPA. The image shows a heatmap of the most DE genes in RNF123-OV cell lines (adjusted *p* < 0.05). (**K**) Integration of DE genes identified in RPPA1 (LN18), RPPA2 (HS683), and RNA-sequencing in RNF123-OV cell lines that are targets of the NF-κB pathway. (**L**) RT-qPCR for *SERPINE1* in A172, HS683, and LN18 cell lines expressing EV1 or RNF123-OV (*t*-test, *** *p* < 0.001). (**M**) Western blot for SerpinE1 in A172, HS683, and LN18 cell lines expressing EV1 or RNF123-OV; low (L) and high (H) exposure times for the same image are shown. (**N**) Correlation analysis of *RNF123* and *SERPINE1* expression using the TCGA dataset from GB tumors (*n* = 145; Spearman’s *r* = −0.27, *p* < 0.001). Error bars represent the mean ± SD from *n* = 3 replicates.

To obtain a model of high RNF123 expression and identified downstream targets, three GB *IDH* WT cell lines were transfected with a plasmid (pCMV6) containing the *RNF123* gene or empty vector (EV1). Clones with high RNF123 expression were selected and confirmed by Western blot (Figure 2A) and real-time quantitative polymerase chain reaction (RT-qPCR) (Figure 2B). RNF123 overexpression (RNF123-OV) resulted in a significant increase of p50 levels (Figure 2A), which decreased proliferation (Figure 2C,D and Figure S2C), colony-forming ability (Figure 2E,F), and invasion (Figure 2G,H) of GB cell lines. In conclusion, RNF123 increased p50 expression and reduced growth in GB cell lines.

### 2.4. Expression of SerpinE1 Is Negatively Associated with RNF123 Expression

To understand the mechanism by which RNF123 decreases GB proliferation and how that may determine outcomes in GB patients, we focused on NF-κB1-targeted genes that were differentially regulated in two RNF123-OV GB cell lines. Therefore, we screened for differentially expressed (DE) genes and proteins, either up- or downregulated, in RNF123-OV cell lines by RNA-sequencing and RPPA, respectively. By RNA-sequencing analysis, 419 genes were found to be DE genes in RNF123-OV cell lines (criteria for inclusion: Log_2_FC < 1 or > 1 with significant adjusted *p*-value < 0.05), of which 263 were downregulated and 156 were upregulated (Figure 2I). Analysis of cell lines with RNF123-OV using the RPPA platform (Log_2_FC > 0.25 or < –0.25, FDR *p* < 0.05) revealed 108 in LN18 (RPPA1) and 49 in HS683 (RPPA2) DE proteins, respectively (Figure 2J). To determine which genes represent known targets of the NF-κB pathway, we downloaded a list of the predicted targets from the http://www.bu.edu/nf-kb/gene-resources/target-genes/. Of the 419 genes identified by RNA-sequencing, only 22 DE genes were predicted to be targets of the NF-κB pathway (Figure 2K). For RPPA analysis only three were commonly predicted to be targets of the NF-κB pathway in both cell lines (Figure 2K). By integrating RNA-sequencing and RPPA screenings, the only consistently and negatively regulated defined target of the NF-κB pathway in RNF123-OV cell lines was SerpinE1 (RPPA1 (Log_2_FC = −0.29 and *p* = 0.0023); RPPA2 (Log_2_FC = −2.05 and *p* = 5.9 × 10^−5^); and RNA-sequencing (Log_2_FC = −1.37 and *p* = 3.97 × 10^−96^; Figure 2K).

The results were validated by analyzing *SERPINE1* expression by RT-qPCR (Figure 2L) and Western blot (Figure 2M) in RNF123-OV GB cell lines compared to control EV1 cell lines. The mRNA and protein expression of SerpinE1 were significantly downregulated in RNF123-OV compared with respective control cell lines (Figure 2L,M). The association between *SERPINE1* and *RNF123* was further validated by in silico analysis. Consistent with our results, *SERPINE1* expression in GB showed a negative correlation with *RNF123* expression using the TCGA dataset (Figure 2N). To summarize, RNF123-OV is able to induce a p50 dependent regulation of NF-κB1-target SerpinE1 in GB cell lines.

### 2.5. SerpinE1 Expression Is Associated with a Poor Prognosis in GB

**Figure 3:**
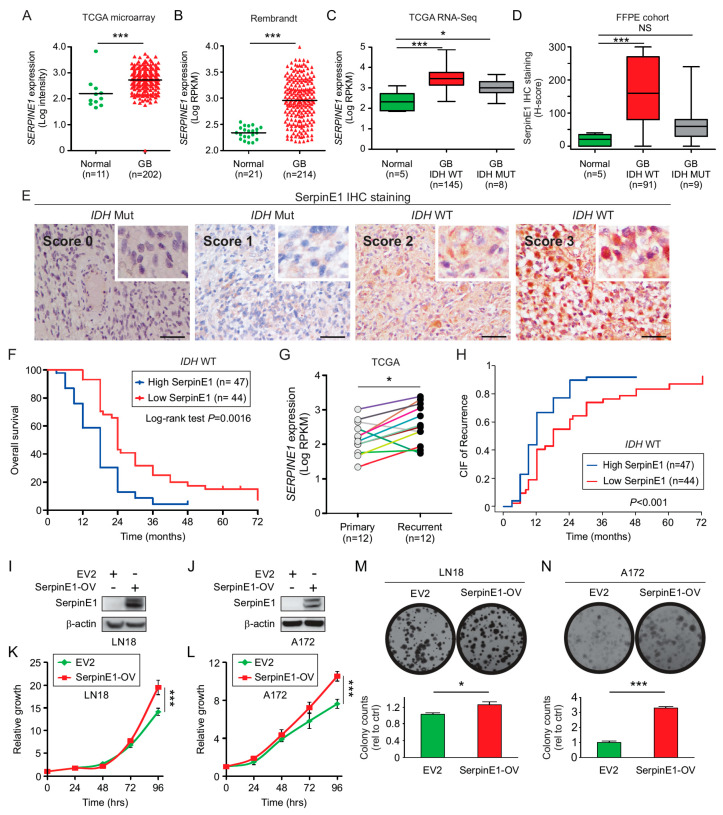
Proliferation and invasion analysis in GB cells with SerpinE1-OV. (**A**) TCGA database analysis of *SERPINE1* in GB tissue (*n* = 202) compared to normal brain tissue (*n* = 11) (*t*-test, *** *p* < 0.001). (**B**) Rembrandt database analysis of *SERPINE1* expression in GB tissue (*n* = 214) compared to normal brain tissue (*n* = 21) (*t*-test, *** *p* < 0.001). (**C**) Boxplot of *SERPINE1* expression in *IDH* WT (*n* = 145) or mutated (*n* = 8) GB patients using the RNA-sequencing TCGA dataset and compared to normal brain tissue (*n* = 5) (Kruskal–Wallis test, Dunn’s post hoc test * *p* < 0.05, *** *p* < 0.001). (**D**) Boxplot of SerpinE1 expression in FFPE samples from normal brain tissue (*n* = 5) and GB tumors with *IDH* WT (*n* = 91) or mutated (*n* = 9) as determined by H-scores (Kruskal–Wallis test, Dunn’s post hoc test *** *p* < 0.001, NS = non-significant). (**E**) Representative IHC images for SerpinE1 staining in FFPE samples showing the scores (0, 1, 2, or 3). Scale bar = 50 μm. (**F**) Kaplan–Meier curves to compare the percentage of survival in GB patients with a low (*n* = 44) versus high (*n* = 47) SerpinE1 expression (log-rank test, *p* = 0.0016). (**G**) *SERPINE1* expression in primary GB tumors (*n* = 12) paired with recurrent tumors (*n* = 12, *t*-test, * *p* < 0.05). (**H**) CIF of recurrent GB patients with low (*n* = 44) versus high (*n* = 47) SerpinE1 expression (*p* < 0.001). (**I**,**J**) Western blot analysis of SerpinE1 in LN18 (**I**) and A172 (**J**) cell lines expressing EV2 or SerpinE1-OV. (**K**,**L**) Proliferation assays in LN18 (**K**) and A172 (**L**) cell lines expressing EV2 or SerpinE1-OV (two-way ANOVA, Bonferroni correction *** *p* < 0.001). (**M**,**N**) LN18 (**M**) and A172 (**N**) cell lines with SerpinE1-OV showed an increased ability to form colonies compared to cell lines expressing control EV2 (*t*-test, * *p* < 0.05; *** *p* < 0.001). Error bars represent the mean ± SD from *n* = 3 replicates.

To evaluate the association of *SERPINE1* expression with GB, two independent microarray datasets (GB TCGA and Rembrandt) were assessed. *SERPINE1* expression was significantly higher in GB tumors than in normal brain tissue (Figure 3A,B). Similarly, the mRNA of *SERPINE1* was assessed in *IDH* WT and mutated GB patients. Only GB patients with *IDH* WT have significantly higher *SERPINE1* expression compared to normal brain tissue (Figure 3C). These results were validated in a larger cohort of normal brain tissue from the GTEX dataset using the GEPIA database (Figure S2B). In agreement with this observation, IHC analysis of an independent cohort of well-annotated clinical specimens (TMA2, containing 100 GB patients and 5 normal brain tissues, Table S2B) also revealed elevated significant expression of SerpinE1 in *IDH* WT GB tumors compared to normal brain tissues (Figure 3D,E). Moreover, patients with high SerpinE1 expression showed a poorer prognosis than GB tumor patients with low SerpinE1 expression (Figure 3F). In a multivariable analysis, both *MGMT* promoter methylation (*p* < 0.02, HR = 1.86, 95% CI 1.12–3.10) and a high SerpinE1 expression were independent factors associated with poor OS (*p* < 0.001, HR = 2.41, 95% CI 1.48–3.92, Table S3A).

To determine the association of *SERPINE1* expression with GB recurrence, *SERPINE1* expression was compared in 12 pairs of patients with recurrent GB using the clinically annotated TCGA mRNA database. Patients with GB recurrence showed significantly elevated *SERPINE1* (Figure 3G). To validate the observation of increased *SERPINE1* mRNA expression in GB recurrence, we performed competing risks regression analysis to evaluate the effect of SerpinE1 on progression-free survival (PFS), taking the competing risk of mortality (or death) into account. CIF showed that *MGMT* promoter methylation (*p* < 0.01, RR = 1.91, 95% CI 1.17–3.11) and high SerpinE1 protein expression were independent factors for predicting PFS (*p* < 0.001, RR = 1.86, 95% CI 1.18–2.95; Table S3B). Similarly, GB patients with a high SerpinE1 protein expression showed an increased CIF of recurrence compared to those patients with low SerpinE1 protein expression (Figure 3H). A high *SERPINE1* mRNA and protein expression was associated with a highly unfavorable prognosis in *IDH* WT GB patients and an increased risk of recurrence.

### 2.6. SerpinE1-OV Promotes Proliferation, Invasion, and Colony-Formation Ability of GB Cells

**Figure 4:**
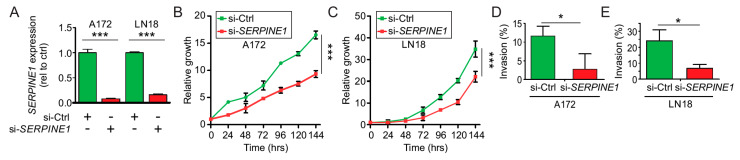
SerpinE1 knockdown reduces GB cell proliferation. (**A**) A172 or LN18 cell lines were transfected with control siRNA (si-Ctrl) or siRNA for SERPINE1 (si-*SERPINE1*) and SERPINE1 expression was analyzed by RT-qPCR (*t*-test, *** *p* < 0.001). (**B**,**C**) Proliferation assays for A172 (**B**) and LN18 (**C**) cell lines transfected with siRNA control (si-Ctrl) or siRNA for *SERPINE1* (si-*SERPINE1*) (two-way ANOVA, Bonferroni correction, *** *p* < 0.001). (**D**,**E**) Invasion assays for A172 and LN18 cell lines transfected with siRNA control (si-Ctrl) or siRNA for *SERPINE1* (si-*SERPINE1*). Shown is the percentage of invasion for A172 (**D**) or LN18 (**E**) (*t*-test, * *p* < 0.05).

As described in Table S3A, SerpinE1 is a prognostic factor in *IDH* WT GB patients and SerpinE1 has been reported to promote tumor growth in different solid tumors. Therefore, we focused on understanding its function in *IDH* WT GB cell lines. Lentivirus carrying *SERPINE1* or the empty vector (EV2) were transduced in two *IDH* WT GB cell lines. Positive clones for *SERPINE1* or the EV2 expression were selected and confirmed by Western blot (Figure 3I,J). To determine the effects of SerpinE1 overexpression (SerpinE1-OV) in *IDH* WT GB cell lines, we performed proliferation and colony-forming assays. Both the proliferation rates (Figure 3K,L) and the number of colony-forming units (Figure 3M,N) were significantly increased in SerpinE1-OV cell lines compared to control EV2 cell lines. To determine whether the increased rates of proliferation observed in the cell lines with SerpinE1-OV are due to reduced apoptosis, PARP1 cleavage and caspase-3 cleavage were analyzed and quantified. No significant changes were observed for PARP1 or caspase-3 cleavage on cell lines with SerpinE1-OV compared to control cells (Figure S2D–F). To further evaluate the importance of SerpinE1 in GB, we transfected *IDH* WT GB cell lines with a siRNA pool against *SERPINE1*. *SERPINE1* expression significantly decreased in A172 and LN18 *IDH* WT GB cell lines (Figure 4A). To validate the results that SerpinE1 has a tumorigenic role in GB as observed in Figure 3K–N, we analyzed cell proliferation and invasion after *SERPINE1* knockdown. Knockdown of *SERPINE1* (si-*SERPINE1*) decreased cell proliferation (Figure 4B,C) and invasion (Figure 4D,E) compared to si-Ctrl-treated cell lines. Thus, confirming the role of SerpinE1 in promoting GB cell growth. In vitro analysis showed that SerpinE1-OV promotes tumor growth and invasion in *IDH* WT GB cell lines; on the contrary *SERPINE1* depletion reduced both cell proliferation and invasion levels.

### 2.7. SerpinE1 Overexpression Rescued the Inhibitory Effects Mediated by RNF123-OV- in GB Cell Lines

**Figure 5:**
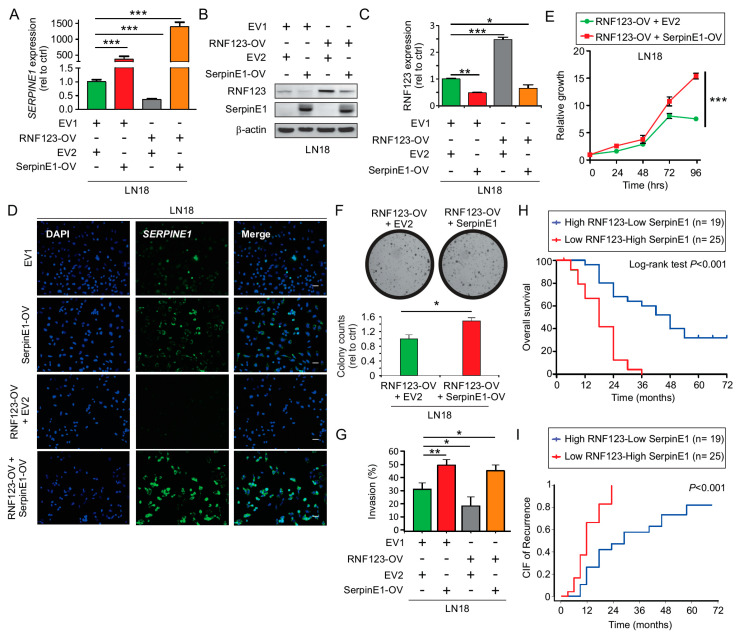
In vitro analysis of SerpinE1 importance in cells with RNF123-OV. (**A**) RT-qPCR for *SERPINE1* in LN18 cell lines with EV1 + EV2, RNF123-OV + EV2, EV1 + SerpinE1-OV, or RNF123-OV and SerpinE1-OV B (one-way ANOVA, Bonferroni correction, *** *p* < 0.001). (**B**) LN18 cell lines with EV1 + EV2, RNF123-OV + EV2, EV1 + SerpinE1-OV, or RNF123-OV and SerpinE1-OV were tested for SerpinE1 and RNF123 expression levels by Western blot. β-actin was used as a loading control. (**C**) Quantification of RNF123 expression analyzed by Western blot in B (one-way ANOVA, Bonferroni correction, * *p* < 0.05, ** *p* < 0.01, and *** *p* < 0.001). (**D**) RNA in situ hybridization analysis of *SERPINE1* in LN18 cell lines with EV1 + EV2, RNF123-OV + EV2, EV1 + SerpinE1-OV, or RNF123-OV and SerpinE1-OV. DAPI was utilized to stain the nuclei (Left). The images are also shown merged (Right). Scale bars: 5 µm. (**E**,**F**) Proliferation (**E**) or colony-forming units (**F**) of LN18 cell lines stably expressing RNF123-OV + EV2 or RNF123-OV + SerpinE1-OV (* *p* < 0.05, *** *p* < 0.001). (**G**) Quantification of the invasion assays was performed in LN18 cell lines stably expressing RNF123-OV + EV2 or RNF123-OV + SerpinE1-OV (one-way ANOVA, * *p* < 0.05, ** *p* < 0.01). (**H**) Kaplan–Meier curves for GB patients expressing high RNF123 and low SerpinE1 (*n* = 19) versus GB patients expressing low RNF123 and high SerpinE1 (*n* = 25) (log-rank test, *p* < 0.001). (**I**) CIF of patients with recurrent GB expressing high RNF123 and low SerpinE1 (*n* = 19) versus those expressing low RNF123 and high SerpinE1 (*n* = 25, *p* < 0.001). Error bars represent the mean ± SD from *n* = 3 replicates.

Based on the suppressive effects that RNF123-OV exerts on cell growth, we tested whether SerpinE1-OV could reverse those suppressive effects. A control (EV2) or *SERPINE1* expressing vector was transfected in LN18 GB cell lines that were either stably expressing a control (EV1) or *RNF123* (RNF123-OV). SerpinE1-OV in LN18-EV1 or LN18-RNF123-OV cell lines was confirmed by RT-qPCR (Figure 5A) and Western blot (Figure 5B). A significant reduction in the levels of RNF123 was determined by Western blot in cell lines with SerpinE1-OV and RNF123-OV (Figure 5B,C). Similar results were observed in HS683 cell lines (Figure S2G). RNA in situ hybridization showed that the mRNA expression of *SERPINE1* was significantly reduced in LN18-RNF123-OV compared to control cell lines. SerpinE1-OV restored the levels of SerpinE1 in the cell lines with RNF123-OV (Figure 5D). We then assessed the effects of SerpinE1-OV on proliferation and colony-formation assays of GB cell lines with RNF123-OV. There was a significant enhancement of proliferation (Figure 5E) and colony-forming units (Figure 5F) in LN18-RNF123-OV/SerpinE1-OV compared to LN18-RNF123-OV/EV2 cell lines, thus confirming that SerpinE1-OV rescued the suppressive effect of RNF123-OV. Additionally, SerpinE1-OV was able to rescue the suppressive effect of RNF123-OV on invasion ability (Figure 5G). Our hypothesis stated that SerpinE1 and RNF123 interaction promotes RNF123 degradation. To test this hypothesis, SerpinE1 was immunoprecipitated from GB cell lysates. The immunoprecipitated complexes were tested for the presence of RNF123 by Western blot. RNF123 was not detected in the immunoprecipitated complexes, suggesting that SerpinE1-mediated effects on RNF123 expression may not be via an interaction between these two proteins (Figure S2H).

### 2.8. Concurrent Low RNF123 and High SerpinE1 Expression Is Associated with a Poor Prognosis

To determine if the negative association between RNF123 and SerpinE1 expression is of prognostic value, our cohort of *IDH* WT GB patients (common patients in TMA1-TMA2, Table S4A) was stratified into two groups: 1) low RNF123 and high SerpinE1 expression and 2) high RNF123 and low SerpinE1 expression (as determined by H-score from IHC analysis in Figure 1D,E and Figure 3D,E, respectively). The low RNF123 and high SerpinE1 group had the worst prognosis (Figure 5H,I). Multivariable analysis revealed that the concurrent presence of low RNF123 and high SerpinE1 expression (*p* < 0.001, HR = 2.93, 95% CI 1.17–5.05, Table S4B) and KPS < 85 (*p* < 0.02, HR = 1.79, 95% CI 1.09–2.92, Table S4B) were independent risk factors associated with poor OS. CIF showed that low RNF123 and high SerpinE1 expression (*p* < 0.001, HR = 3.56, 95% CI 1.61–7.83, Table S5) and KPS < 85 (*p* < 0.04, HR = 2.04, 95% CI 1.04–4.02, Table S4B) were independent risk factors associated with increased PFS (Table S5). Finally, we performed a multivariable analysis considering RNF123 and SerpinE1 as single variables with other clinical variables to determine their importance as a predictor prognostic factor for OS. High SerpinE1 expression (*p* < 0.01, HR = 2.01, 95% CI 1.18–3.44, Table S4B) and KPS < 85 (*p* < 0.02, HR = 2.27, 95% CI 1.14–4.50, Table S6) were independent prognostic factors associated with poor OS.

### 2.9. MiR-155 Suppresses RNF123 Expression in GB and Is Associated with a Poor Prognosis

**Figure 6:**
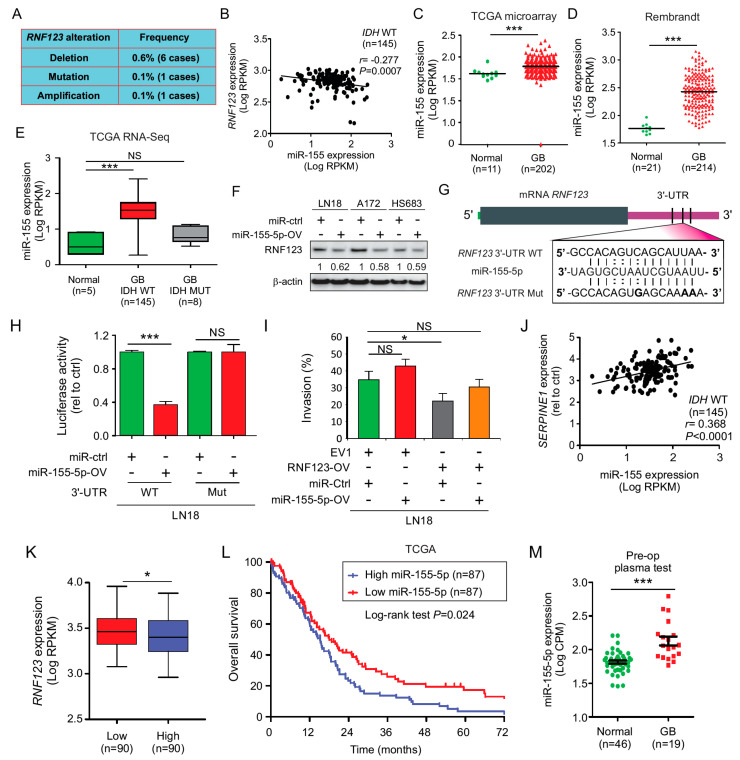
MiR-155-5p decreased *RNF123* expression and gave a poor prognosis in GB patients. (**A**) TCGA analysis of a merged cohort of low-grade glioma and GB for copy number variations and mutations. The frequency of *RNF123* alteration is 0.8% of a total of 1084 patients. (**B**) Correlation analysis of miR-155-5p and *RNF123* expression using TCGA dataset from GB tumors (*n* = 145; Pearson *r* = −0.277, *p* = 0.0007). (**C**) TCGA database analysis of miR-155 in GB tissue (*n* = 202) compared to normal brain tissue (*n* = 11) (*t*-test, *** *p* < 0.001). (**D**) Rembrandt database analysis of miR-155 expression in GB tissue (*n* = 214) compared to normal brain tissue (*n* = 21) (*t*-test, *** *p* < 0.001). (**E**) TCGA database analysis of RNA-sequencing data for miR-155 in *IDH* WT (*n* = 145) or mutated (*n* = 8) GB tissue compared to normal brain tissue (*n* = 5) (one-way ANOVA, *** *p* < 0.001, NS = non-significant). (**F**) LN18, A172, and HS683 cell lines were transfected with pre-miR-155-5p (miR-155-5p-OV) or miR control (miR-Ctrl) and RNF123 expression was quantified by Western blot. (**G**) miR-155-5p sequence aligned with human *RNF123* WT 3′-UTR (WT) and *RNF123* Mutant 3′-UTR (Mut) sequences. (**H**) Luciferase reporter activity assay to determine the effect of miR-155-5p on 3′-UTR of *RNF123* using human *RNF123* 3′-UTR (WT) and *RNF123* Mutant 3′-UTR (Mut) sequences cloned in RenSP vector (*t*-test, NS = non-significant, *** *p* < 0.001). (**I**) Percentage of invasion of LN18 cell lines with miR-155-5p-OV, RNF123-OV, or both compared to control cell lines (one-way ANOVA, * *p* < 0.05, NS = non-significant). (**J**) Correlation analysis of miR-155-5p and *SERPINE1* expression using TCGA dataset from GB tumors (*n* = 145; Pearson *r* = 0.368, *p* < 0.0001). (**K**) GB patients from the TCGA database were split into low (*n* = 90) and high (*n* = 90) miR-155-5p expression and analyzed for RNF123 expression (*t*-test, * *p* < 0.05). (**L**) Kaplan–Meier curves for the OS of GB patients expressing low (*n* = 87) versus high (*n* = 87) miR-155-5p (log-rank test, *p* = 0.024). (**M**) Dot plot to determine miR-155-5p expression in pre-operative plasma from GB patients (*n* = 19) and plasma of healthy controls (*n* = 46) (*t*-test, ** *p* = 0.024). Error bars represent mean ± SD from replicates (*n* = 3).

To comprehend the underlying mechanism behind the regulation of *RNF123* expression in GB, we assessed for mutations and copy number variation (deletions or amplifications) in *RNF123* gene using the GB TCGA database. The results showed that GB tumors have a very low frequency of mutations and copy number variations in *RNF123* gene (Figure 6A), suggesting that RNF123 expression is not regulated by a genomic functional event. In a previous report from our group, we found that miR-155-5p specifically targets RNF123 and reduces its expression in melanoma [9]. MiR-155-5p has been reported as an oncogenic microRNA (miR) in GB [9,41,42]. To determine a role for miR-155-5p in modulating RNF123 expression GB TCGA dataset was assessed. MiR-155-5p showed a negative association with RNF123 in *IDH* WT GB patients (Figure 6B), and miR-155-5p expression was elevated in GB tumors compared to normal brain tissues (Figure 6C). Similar results were obtained using the Rembrandt database (Figure 6D). *IDH* WT GB patients have increased miR-155-5p expression compared to normal brain tissue (Figure 6E). MiRs recognize and bind to specific sequences in the mRNA, thus miRs work as regulators of mRNA expression. To determine the regulatory effects of miR-155-5p on RNF123 expression, miR-155-5p was overexpressed (miR-155-5p-OV) in three *IDH* WT GB cell lines. Western blot analysis revealed a decreased expression of RNF123 in cell lines with miR-155-5p-OV (Figure 6F). To determine if miR-155-5p targets RNF123, we cotransfected miR-155-5p and a luciferase reporter constructs bearing either the WT or mutated 3′-UTR of RNF123 (as indicated in Figure 6G). MiR-155-5p decreased the luciferase expression only when cotransfected with the WT 3′-UTR but not with the mutant 3′-UTR, indicating that miR-155-5p specifically targets the 3′-UTR of RNF123 in GB cell lines (Figure 6H). MiR-155-5p-OV did not affect the ability of GB cell lines to invade (Figure 6I), but partially rescue the effects of RNF123-OV by targeting the endogenous RNF123 (Figure 6I). Using the TCGA dataset, GB patients with a high and low miR-155 expression were evaluated for RNF123 expression. Low miR-155 expression was associated with high RNF123 expression in GB tumors (Figure 6K). Furthermore, patients with high miR-155 expression had a poorer prognosis than those with low expression (Figure 6L). The expression of miR-155 and *SERPINE1* was positively correlated (Figure 6J), showing that the negative regulatory effect of miR-155-5p on *RNF123* may indirectly promote SerpinE1 expression; in addition, SerpinE1 negatively regulates RNF123 expression.

GB patients were molecularly classified by TCGA into five subtypes (normal, classical, neural, proneural, and mesenchymal) based on mRNA expression profiles [2,5]. Using this classification and TCGA datasets for GB patients, RNF123 expression was decreased in patients with the mesenchymal and neural subtypes compared to those with the normal subtype (Figure S3A). GB patients with the mesenchymal subtype had higher *SERPINE1* expression than those with any other subtypes (Figure S3B). In all GB subtypes, miR-155 expression was increased compared to normal brain tissues (Figure S3C), with the mesenchymal subtype showing the highest expression of miR-155. These results confirm a strong interrelation driving aggressive GB tumors and support the finding that increased miR-155-5p and SerpinE1 decreased RNF123 expression.

To assess the value of miR-155-5p as a prognostic marker, preoperative blood samples of GB patients were analyzed and compared with blood samples from healthy individuals. We utilized the HTG EdgeSeq miR whole transcriptome assay platform to analyze the normal and GB plasma samples. Higher expression of miR-155-5p was observed in preoperative blood samples from GB patients than in normal blood samples (Figure 6M). These results suggested a clinical utility of miR-155-5p as a potential blood cell-free biomarker for GB patient assessment using a minimal invasive assay.

## 3. Discussion

Ubiquitin E3 ligases have a critical role in normal human cells in regulating ubiquitination and degradation of proteins, an essential metabolic function for survival. The function of E3 ligases depends on their catalytic activity as well as substrate specificity. Recently, we demonstrated that RNF123 in the context of tumor progression was downregulated in metastatic melanoma functioning as a tumor suppressor gene [43]. E3 ligases play a critical role in GB to keep the homeostasis of different cellular functions and oncogenic pathways in this highly aggressive primary brain tumor. There is growing evidence showing the importance of E3-ligases in affecting specific pathways in GB. In the present study, the E3-ligase RNF123 showed specificity in recognizing and processing NF-κB1 into p50. No changes were observed in p27 expression, a key cell cycle regulator, in GB tumors with an increase in RNF123 expression, as shown in previous reports [17,44]. This finding is interesting as we have previously demonstrated that RNF123 is a critical factor recognizing p50 but not p27 in melanoma [9]. The best explanation for this observation is that p27 expression may be very low in GB cells with high doubling rates. Also, p27 is a critical factor controlling the cell cycle and can be modified by multiple factors including other E3-ligases [45] in GB, as well as stabilized by ubiquitination as recently shown [46].

The NF-κB pathway is constitutively activated in GB, whereby many central oncogenic pathways converge through the NF-κB system [15]. The NF-κB pathway promotes the malignant phenotype and mesenchymal transition of the mesenchymal subtype of GB [47]. In the present study, we demonstrated that the aberrant expression of different components of the miR-155-5p-RNF123-NF-κB1-p50-SerpinE1 pathway. In summary, RNF123 is significantly downregulated in GB tumors, as shown by transcriptomic analyses using publicly available databases, with a significant and particularly prominent reduction of RNF123 in GB patient bearing *IDH* WT. These results were further confirmed by IHC and H-scores using our independent cohort of GB patients. In GB cell lines, RNF123-OV reduced cell proliferation and invasion by increasing p50 expression. Integrative analysis of DE genes showed that *SERPINE1* was the major NF-κB1 target affected by RNF123-OV in GB. SerpinE1 was an oncogenic factor upregulated in *IDH* WT GB tumors with low RNF123 expression. However, little is known on the effect of SerpinE1 on GB cell growth and invasion. SerpinE1 is a secreted factor with protease activity it is expected to have major implications in remodeling the extracellular matrix (ECM), thus promoting cell invasion. There are limited studies on the role of SerpinE1 and GB growth and invasion. Further, studies are needed to demonstrate how enhanced SerpinE1 is released and accumulates outside the cells and how this influences the cell growth and invasion of GB cells in a tumor microenvironment. GBs are difficult to treat in advance stages because of their propensity to invade normal brain tissues and spread to different areas of the brain. SerpinE1 may be a potential target in adjuvant treatment of GB patients having surgical resection or other therapies.

As we demonstrated in this study, genomic modifications do not impact RNF123 expression. To better explain RNF123 downregulation, we demonstrate that elevated SerpinE1 and miR-155-5p expression modulated RNF123 levels. While miR-155-5p directly targeted the 3′-UTR mRNA of *RNF123* and reduced RNF123 expression, SerpinE1 overexpression reduced RNF123 expression however, the mechanism is not through a direct interaction between the SerpinE1 and RNF123. Further studies are needed to demonstrate the pathways affected by SerpinE1 overexpression that regulate RNF123 expression in GB. Understanding this event would be highly important to determine key factors that may be associated with prognosis in GB and are regulated by SerpinE1.

RNF123 exerts major control on NF-κB1 processing and RNF123 downregulation reduces the formation of p50 dimers. The reduction in p50 dimers removes the tight control on the transcription of NF-κB1 target genes such as SerpinE1. There are limited studies reported on SerpinE1 as related to GB progression and patient outcomes. SerpinE1 elevated expression has been associated with multiple types of cancers and their progression as well as benign diseases. Our study demonstrated the importance of RNF123 in modulating the activation of the SerpinE1 and relation to GB tumor progression. Further studies are needed to characterize the downstream pathways activated by SerpinE1 that promote *IDH* WT GB tumor progression.

Our studies demonstrated the role of miR-155-5p in blocking RNF123 expression in GB cells, an observation that we also described in cutaneous melanoma cells [9]. Recently, Wu X. et al. reported a role for miR-155-5p in controlling GB mesenchymal transition [41]. The mesenchymal GB subtype has a poor prognosis and is resistant to therapies [48]. Interestingly, the proposed components of the pathway are also aberrantly expressed in patients with the mesenchymal subtype of GB compared to the other subtypes. A recent study using RPPA data from TCGA demonstrated characteristic unique proteomic signatures of GB subtypes and showed an association between the mesenchymal subtype and *SERPINE1*, thus supporting our results showing an increased mRNA expression of SerpinE1 in GB with the mesenchymal subtype [49].

Our study demonstrated that elevated circulating cell-free miR-155-5p levels are detectable in plasma samples of pre-operative GB patients. This suggested that this key elevated regulatory miR may have a role as a diagnostic blood biomarker. This is an important finding as the elevation of a specific circulating cell-free miR in blood can be a surrogate of RNF123 downregulation of GB during progression. There are very limited blood biomarkers of GB detection and progression. The advantage of circulating cell-free miR as blood biomarkers is that they are more stable in blood than cell-free DNA. Also, cell-free DNA has not been very reliable in GB assessment to date. Further evaluation of circulating cell-free miR-155-5p in GB patients will determine its potential clinical utility in diagnosis and during treatment.

Low RNF123 expression, as well as high SerpinE1 and high miR-155, were strong and independent predictive factors of poor prognosis in *IDH* WT GB patients. High SerpinE1 expression or low RNF123 expression increased the risk of progression as shown through CIF analysis and are independent factors associated with OS in a multivariable analysis in *IDH* WT GB patients. These analyses demonstrated the importance of both RNF123 and SerpinE1 in GB progression. Additionally, concurrent low RNF123 and high SerpinE1 expression was an independent prognostic factor associated with OS and enhancement of recurrence. The in vitro studies on the mechanism of RNF123 and SerpinE1 support the clinical findings of GB. Future studies are needed to determine the temporal expression of these two genes during the progression of the early stages of glioma to GB. The real-time analysis of these two genes status on resected GB tumors may help to better triage patients based on their individual risk of progression onto appropriate therapeutic intervention.

## 4. Materials and Methods

### 4.1. Ethics Approval and Consent to Participate

This study followed the principles in the Declaration of Helsinki. All human samples and clinical information for this study were obtained according to the protocol guidelines approved by the Saint John’s Health Center (SJHC)/John Wayne Cancer Institute (JWCI) Western Institutional Review Board and Human Research Ethics Committee of Huashan Hospital, Fudan University, Shanghai, China. Informed consent was obtained from all participants. FFPE tissues of different grade glioma and normal brain tissue were provided by the Department of Neurosurgery, Huashan Hospital, Shanghai (GB, *n* = 100; normal brain tissue, *n* = 5) under the protocols: KY2016-400 and KY2015-256. Plasma was collected as previously described [50] and obtained under the SJHC/JWCI protocol: MORD-RTPCR-0995.

### 4.2. Clinical Characteristics of Patients

The clinical data was obtained from the medical records of patients who underwent surgical resection. Tumors samples from the glioma tissue bank were collected from 2011 to 2013 at Huashan Hospital, Fudan University, Shanghai, China. The clinical data, including demographic data, tumor characteristics, treatment strategy, and survival data, were retrospectively reviewed. Two TMAs were used in this study. The TMA1 was used to stain RNF123 and the TMA2 to stain SerpinE1. Only 85 patients overlap in both TMAs. The mean overall survival of the patients included in the TMA1 and TMA2 was 26 months. The *IDH1* and *IDH2* genes mutation status was detected by PCR. MGMT promoter methylation status was determined by methylation-specific PCR (MSPCR). All the patients received a standard of care treatment that consists of radiotherapy and concomitant adjuvant chemotherapy with temozolomide. All the patients included were evaluated for KPS ranging from 70 to 100 on a scale of 0–100.

### 4.3. Cell Lines

Human glioma cell lines A172 (RRID: CVCL_0131), LN18 (RRID: CVCL_0392), and HS683 (RRID: CVCL_0844) were obtained from the American Type Culture Collection. Cell lines were cultured in DMEM, Leibovitz’s L-15 Medium (L-15) and EMEM, (all medium purchased from ATCC, Manassas, VA, USA) and supplemented with 10% fetal bovine serum at 37 °C with 5% CO_2_. All human cell lines have been authenticated using STR profiling within the last three years. All experiments were performed with mycoplasma-free tested cell lines.

### 4.4. Reverse-Phase Protein Array Analysis

Protein lysate from GB cell lines (LN18 and HS683) stably transfected with either Myc-tagged RNF123 or empty-vector (OriGene) was extracted as previously described [9], and reverse-phase protein array (RPPA) analysis was performed by the CCSG-supported RPPA Core Facility at the University of Texas MD Anderson Cancer Center [9,51]. A list of the Abs (*n* = 305) tested can be accessed from https://www.mdanderson.org/research/research-resources/core-facilities/functional-proteomics-rppa-core.html. Heatmaps plots of the data DE genes were shown. Differences in protein expression between groups were determined using a student’s *t*-test with a two-sided *p*-value < 0.05.

### 4.5. Immunohistochemistry for Tissue Microarrays

FFPE TMA including 100 GB patients and 5 normal brain tissues were obtained from the Department of Neurosurgery, Huashan Hospital, Shanghai. All the patients were clinically well-annotated with greater than 5 years of follow-up. IHC was performed as previously described [9], but using the following Abs: mouse anti-human RNF123 monoclonal Ab (mAb) (1:50 dilution, ab57549; Abcam), rabbit anti-human NF-κB1 p50 polyclonal IgG Ab (1:200 dilution, sc-114; Santa Cruz Biotechnology, CA, USA), or mouse anti-human p27 (CDKN1B) mAb (1:100 dilution, 610241; BD Biosciences), rabbit anti-PAI-1(SerpinE1) polyclonal IgG Ab (1:100, HPA050039; Sigma-Aldrich Corp, St. Louis, MO, USA).

### 4.6. Stable Clones Overexpressing RNF123

To establish RNF123 overexpressing clones, LN18, A172, and HS683 cell lines (5 × 10^5^ cells in 60 mm dishes) (Corning, NY, USA) were transfected with Myc-tagged *RNF123* vector (OriGene, Rockville, MD) using the jetPRIME transfection reagent. The empty vector pCMV6 (EV1) was used to transfect cell lines as negative controls. Positive clones were selected using Geneticin (500 µg/mL, Life Technologies, Carlsbad, CA, USA) and RNF123-OV was confirmed by Western blot and RT-qPCR. All experiments that involved RNF123-OV clones were performed within ten passages after their establishment.

### 4.7. Stable Clones Overexpressing SERPINE1

To establish stable SerpinE1-OV clones, LN18 and A172 cell lines (5 × 10^3^ cells in 24-well culture plates were transduced with *SERPINE1* vector using lentivirus particles (Cat# LPP-F0606-Lv121-050; Genecopoeia, Rockville, MD, USA). The empty vector pReceiver Lv-121 (EV2) was used to transfect cell lines (LN18 and A172) as negative controls (Cat# LPP-NEG-Lv121-050; Genecopoeia). Positive clones were selected using Puromycin (1 µg/mL, Life Technologies) and SerpinE1-OV was confirmed by Western blot and RT-qPCR. All experiments that involved SerpinE1-OV were performed within ten passages after their establishment.

### 4.8. Bioinformatics Analysis

The Cancer Genome Atlas (TCGA) data sets available for GB tumors for mRNA expression, miR expression, and clinical information was obtained in November 2019 (https://www.cbioportal.org/). TCGA data available at the betastasis database (http://www.betastasis.org) was also utilized to predict prognosis and gene expression. The NFκB-target gene list was obtained from (http://www.bu.edu/nf-kb/gene-resources/target-genes/). The GEPIA database was assessed for RNF123 and SerpinE1 expression (http://gepia.cancer-pku.cn/).

### 4.9. Data Availability

The data generated and discussed in this publication have been deposited in the NCBI Gene Expression Omnibus and are accessible through GEO Series accession number GSE131402 (https://www.ncbi.nlm.nih.gov/geo/query/acc.cgi?acc=GSE131402).

### 4.10. Information Available in Supplementary Data

The following items were included in Supplementary Material and Methods: (1) Reagents, (2) RNA extraction, RNA sequencing, and RT-qPCR analysis, (3) MiR transfection, (4) Luminescent reporter gene transfections and luciferase assay, (5) Small interference RNA for *SERPINE1*, (6) HTG miR profiling, (7) RNA in situ hybridization, (8) Cell viability and colony formation assays, (9) Cell migration and invasion assays, (10) Western blot (Detailed information can be found at Figure S4), (11) Biostatistical analysis.

## 5. Conclusions

RNF123 is a critical factor controlling p50 levels. RNF123 is expressed in normal brain tissue and in aggressive GB *IDH* WT tumors RNF123 expression decreased. On this downregulation of RNF123 the number of p50–p50 dimers decreased and hence their suppressive activity on transcription of NF-κB1 target genes. Consequently, the expression of specific NF-κB1 targets such as SerpinE1 increased. Hence, increased SerpinE1 expression enhanced *IDH* WT GB cell lines growth and invasion. In GB *IDH* WT tumors the levels of RNF123 were decreased by increased expression of miR-155-5p and SerpinE1 (see Graphical Abstract). Of clinical relevance, both low RNF123 and high SerpinE1 were prognostic factors to predict OS and an increased risk of recurrence. MiR-155-5p is prognostic factor that can be detected in the blood samples of GB patients.

## Supplementary Materials

The following are available online at https://www.mdpi.com/2072-6694/12/5/1081/s1, Supplementary Materials and Methods: Supplementary data; Figure S1: Immunohistochemistry analysis of p50 and p27 in GB, Figure S2: SerpinE1 and RNF123 expression in GB. Role of SerpinE1 in modulating apoptosis and controlling RNF123 expression, Figure S3: GB with mesenchymal subtype showed increased expression of *SERPINE1*, Figure S4: Western blot uncropped images used in the manuscript (Opt Densitometry of all western blot can be found at supplementary excel), Table S1A: Clinical pathological features of GB patients from TMA1, Table S1B: Univariate and Multivariable analysis for the association between RNF123 expression and overall survival, Table S2A: Univariate and Multivariable Analysis for the Association between RNF123 expression and Recurrence-free Survival using Competing Risk Regression, Table S2B: Clinical pathological features of GB patients from TMA2, Table S3A: Univariate and Multivariable Analysis for the Association Between SerpinE1 expression and Overall Survival, Table S3B: Univariate and Multivariable Analysis for the Association between SerpinE1 expression and Recurrence-free Survival using Competing Risk Regression, Table S4A: Clinical pathological features of GB patients from TMA1 and TMA2, Table S4B: Univariate and Multivariable Analysis for the Association Between combined RNF123/SerpinE1 expression and Overall Survival, Table S5: Univariate and Multivariable Analysis for the Association between combined RNF123/SerpinE1 expression and Recurrence-free Survival using Competing Risk Regression, Table S6: Univariate and Multivariable Analysis for the Association Between combined RNF123/SerpinE1 expression and Overall Survival.

## Abbreviations

3′-UTR	3′-Untranslated Region
Abs	Antibodies
B2MG	Beta-2-Microglobulin
CI	Confidence Interval
CIF	Cumulative Incidence Function
EV	Empty Vector 1 and 2
HR	Hazard Risk
FFPE	Formalin-Fixed Paraffin Embedded
GB	Glioblastoma
IDH	Isocitrate Dehydrogenase 1 and 2 Gene
IHC	Immunohistochemistry
KPS	Karnofsky Performance Score
NF-κB	Nuclear Factor kappa-Light-Chain-Enhancer of Activated B Cells
NS	Non-Significant
MiR	MicroRNA
MGMT	Methyl guanine methyltransferase
OS	Overall Survival
PBS	Phosphate Buffer Saline
PFS	Progression-Free Survival
RPPA	Reverse-Phase Protein Arrays
RR	Relative Risk
RT-qPCR	Real-time quantitative polymerase chain reaction
SD	Standard Deviation
TCGA	The Cancer Genome Atlas Project
TMA	Tissue Microarray
UPS	Ubiquitin-proteasome system
WT	Wild Type
WHO	World Health Organization
