# Supplementary material for: Downregulation of the Ubiquitin-E3 Ligase RNF123 Promotes Upregulation of the NF-κB1 Target SerpinE1 in Aggressive Glioblastoma Tumors
Source: Cancers (Basel). 2020 Apr 27;12(5):1081. doi: 10.3390/cancers12051081 (PMC7281601; doi:10.3390/cancers12051081)
Supplement: Supplementary file 1 [file cancers-12-01081-s001.zip › cancers-769385 - supplementary - final/cancers-769385 - supplementary - final.pdf]

Supplementary Material and Methods

Downregulation of the Ubiquitin-E3 Ligase RNF123 promotes upregulation of the NF-κB1 target SerpinE1 in aggressive glioblastoma tumors

Xiaowen Wang, Matias A. Bustos, Xiaoqing Zhang, Romela Irene Ramos, Cong Tan, Yuuki Iida, Shu-Ching Chang, Matthew P. Salomon, Kevin Tran, Rebecca Gentry, Yelena Kravtsova-Ivantsiv, Daniel F. Kelly, Gordon B. Mills, Aaron Ciechanover, Ying Mao and Dave S.B. Hoon

1. Reagents

Dulbecco's Modified Eagle's Medium (DMEM), Leibovitz's L-15 Medium (L-15) and Eagle's Minimum Essential Medium (EMEM) were purchased from ATCC (Manassas, VA). The cell extraction buffer and Pierce BCA assay kit were acquired from Thermo Fisher Scientific (Canoga Park, CA). Quant-iT RiboGreen RNA assay kit and NuPAGE Novex 4-12% and 10% gels were purchased from Life Technologies (Carlsbad, CA, USA). CellTiter-Glo® Luminescent Cell Viability Assay was from Promega (Madison, WI, USA). The Perfecta qPCR Toughmix Rox, Green fastmix perfecta-iQ, Green supermix perfecta-iQ, Cell striper (Non enzymatic dissociation solution), Jet-PRIME™ Transfection Reagent, and medium HEPES 1M solution were all purchased from VWR International LLC (Radnor, PA, USA). TRI-Reagent was obtained from Molecular Research Center, Inc. (Cincinnati, OH, USA). RNA-Solv Reagent was from Omega Bio-Tek (Norcross, GA). Hsa-RNU6, perfecta universal PCR primers, and qScriptTM cDNA Synthesis kit were all purchased from Quanta Biosciences Inc. (Gaithersburg, MD). The RNAscope® Fluorescent Multiplex reagent kit and probes were purchased from ACD (Newark, CA, USA). Cultrex® BME Cell Invasion Assay were purchased from Trevelgen (Gaithersburg, MD, USA). Corning® BioCoat™ Control Inserts with an 8.0μm PET Membrane in two 24 Well Plates were from Corning (Corning, NY, USA). Puromycin Dihydrochloride, Pre-miR miR Precursor (hsa-miR-155-5p), and Pre-miR™ miR Precursor Negative Control were purchased from ThermoFisher Scientific (Canoga Park, CA, USA). The LightSwitch Luciferase Assay Kit was purchased from Active Motif (Carlsbad, CA, USA).

2. RNA extraction, RNA sequencing and RT-qPCR analysis

RNA extractions of the LN18 cells with RNF123-OV or expressing the negative control vector for RNA sequencing were performed as previously described [1,2]. Total RNA isolation from the cell lines was performed using the Direct-zol RNA MiniPrep kit (Zymo Research, Irvine, CA, USA), according to the manufacturer’s instructions.

RT-qPCR for mRNA and miR was performed as previously described [1,2]. Annealing temperatures for each primer were as follows: *RNF123* (60ºC), *SERPINE1* (55ºC), and miR-155-5p (60ºC). Specific primers were used for the qPCR reactions and these assays were performed as previously described [1,2]. Primer sequences were as follows: *RNF123*, 5′-GTGGGTGTCTCCGATGATGTC-3′ (forward), 5′-CAAGGATGTCCTTCCTCCTCTT-3′ (reverse); *SERPINE1*, 5′-GCCCGATGGCCATTACTACGACATCCTG-3′ (forward), 5′-GGAAAGGCAACATGACC-3′ (reverse). Quantitative expression was referenced by human *SDHA* (Succinate Dehydrogenase Complex, Subunit A) or *B2MG* expression (beta-2-microglobulin): *SDHA*, 5′-TCAGCATGCAGAAGTCAAT-3′ (forward), 5′-GAACGTCTTCAGGTGCTTT-3′ (reverse); *B2MG* 5′-TGTCACAGCCCAAGATAG-3′ (forward), 5′-CAAGCAGCAGAATTTGGAA-3′ (reverse). Quantitative expression of miR-155-5p was then analyzed using a gene-specific primers and referenced to RNU6 expression. Relative gene expression was analyzed using the ΔΔCq Calculation Method from the Cq (quantification cycle) values.

3. MiR transfection

GB cells (2 × 10[5]) were seeded in a 6-well plate and were transfected with 30 nM of miR-155-5p precursor or miR negative control using the jetPRIME transfection reagent. RNA and protein extraction were then performed 48 h after transfection, and miR-155-5p overexpression was validated using RT-qPCR [1,2].

4. Luminescent reporter gene transfections and luciferase assay

GB cells (2 × 10[4], LN18) were seeded into a 96-well plate and then co-transfected with: (1) 30 nM of miR-155-5p precursor and 100 ng of *RNF123* WT 3′-untranslated region (3′-UTR) reporter vector; 2) 30 nM of miR-155-5p precursor and 100 ng of *RNF123* Mutant 3′-UTR reporter vector;) 30 nM of miR negative control precursor and 100 ng of *RNF123* WT 3′-untranslated region (3′-UTR) reporter vector, or (2) 30 nM of miR negative control precursor and 100 ng of *RNF123* Mutant 3′-UTR reporter vector. After 24 h incubation, the LightSwitch Luciferase Assay Reagent was added to each well, and the luciferase signal intensity was assessed by the GloMax-Multi Detection System (Promega, Madison, WI, USA).

5. Small interference RNA for SERPINE1

GB cells (2 × 10[5]) were transfected with 10 nM ON-TARGET plus SMARTpool siRNA for human *SERPINE1* (Dharmacon, Lafayette, CO) using jetPRIME transfection reagent. The following siRNA were used for *SERPINE1* (si-RNA1: Sense 5'-GCCACCAACUUCGGAGUAATT-3'; Antisense 5'-UUACUCCGAAGUUGGUGGCCT-3'); (si-RNA2: Sense 5'-GGACUUCUCAGAGGUGGAATT-3'; Antisense 5'-UUCCACCUCUGAGAAGUCCTT-3') and for control (siRNA: Sense 5'-UUCUCCGAACGUGUCACGUTT-3'; Antisense 5'-ACGUGACACGUUCGGAGAATT-3'). Gene expression was validated 48 h after transfection by western blot or RT-qPCR.

6. HTG miR profiling

For each plasma sample, miR profiles were generated using the HTG EdgeSeq miR Whole Transcriptome Assay (HTG Molecular Diagnostics Inc., Tucson AZ). A total of 19 GB and 46 non-cancer control samples from healthy donors were analyzed using 50 µL of plasma per sample following the manufactures recommendations. HTG Molecular Parsing Software was then used to analyze FASTQ files to generate raw counts for 2083 miRs per sample. Normalization counts for the GB and normal plasma samples were performed using a generalized linear modeling approach implemented in the DESeq2 Bioconductor package [3]. Only DE miRs with a significance threshold of FDR $p< 0.05$ and FC>1 were included.

7. RNA in situ hybridization

GB cells (LN18 and HS683, ~2 × 10[3]) with RNF123-OV and negative empty vector control overexpression were seeded in 8-well culture chamber slide and left to grow for 24 h. On the next day, cells were fixed, dehydrated, and pre-treated according to the manufacturer's instructions (https://acdbio.com/manual-assays-rnascope). The cells were then stained with the Hs-*SERPINE1* probe (#555961) using the RNA-scope Multiplex Fluorescent Kit V2 (ACD, Newark, CA, USA) according to the manufacturer's instructions. The cells were stained with DAPI (ACD, Newark, CA, USA). Positive and negative control probe were also applied in all of the samples according to the manufacturer's instructions. Representative images were taken using a Nikon Eclipse Ti microscope and NIS elements software (Nikon).

8. Cell viability and colony-formation assays

Cells (2.5 x 10[3]) were cultured in a 96-well plate (Thermo Fisher Scientific, Waltham, MA, USA), and the number of viable cells were assessed every 24 h using Cell Titer-Glo Luminescent Cell Viability Assay (Promega, Madison, WI, USA) according to the manufacturer's instructions. For colony-forming assays, cells (2 × 10[3]) were seeded into a 6-well plate. After 7-10 days of incubation,

the cell colonies were fixed with 100% methanol and stained with 0.3% crystal violet solution. The cell colonies were then counted using ImageJ software (http://imagej.nih.gov/ij/).

9. Cell migration and invasion assays

For the cell invasion assays, each well utilized was pre-coasted with a BME membrane. Cells (5 × 10^4 cells/well) were added and incubated for 24 h. The medium was then removed and the cells were detached using a cell dissociation solution containing calcein-AM. The relative fluorescence units (RFUs) were assessed by the GloMax-Multi Detection System (Promega). To determine the percent of invasion the total RFUs were then compared to a standard curve to convert RFUs to a cell number.

10. Western blot

Protein extraction was performed as previously described [1,2]. Briefly, five to ten μg of proteins were separated under 4-12% or 10% SDS/PAGE for at least 2 h and electrotransferred for 1 hr. The wet transfer was conducted at 100V on a PDVF membrane. Nonspecific binding of proteins on the membranes were blocked with 5% skim milk for 1 hr. at RT and then immunoblotted ON at 4°C with primary antibodies. After immunoblotting, the membranes were washed three times with Tris-Buffered Saline containing 0.1% Tween-20 (TBS-T), followed by 1 hr incubation with secondary antibodies. The membranes were washed three times with TBS-T and immunoreactive bands were visualized with the chemiluminescent reagent and acquired on an ECL imager (Thermo Fisher Scientific). The protein bands densities were quantified by ImageJ software. The following primary and secondary antibodies (Abs) were used: mouse anti-human RNF123 (KPC1) Ab (1:300, #ab57549; Abcam, Cambridge, MA), mouse anti-Myc-tag Ab (1:1000, #05-724; Millipore, Billerica, MA), mouse anti-Flag Ab (1:1000, #TA50011-100; OriGene), mouse anti-human β-actin Ab (1:10,000, #A5441; Sigma-Aldrich), rabbit anti-mouse PAI-1 (SERPINE1) Ab (1:500, #MA5-17171; ThermoFisher Scientific, Canoga Park, NY); rabbit anti-human cleaved PARP1 antibody [E51] (#ab32064); rabbit anti-human cleaved caspase-3 antibody (#ab32042); sheep anti-mouse Ab (1:4000, #NA931; GE Healthcare, Pittsburgh, PA) or donkey anti-rabbit Ab (1:4000, #NA934; GE Healthcare)). Immunoreactive bands were visualized with the SuperSignal West Femto Maximum Sensitivity Substrate (Life Technologies), and the densities of protein bands were quantified using ImageJ software (http://imagej.nih.gov/ij/).

11. Biostatistical analysis

Categorical variables were assessed by using the χ^2 test, Fisher's exact test. The data normal distribution was tested using the Kolmogorov–Smirnov test. The parametric student's *t*-test was used for normally distributed data and the non-parametric Wilcoxon rank-sum test was used for non-normally distributed data. According to the data distribution, a one-way ANOVA test or the Kruskal-Wallis test was then applied with the post hoc test (Tukey, Dunnet's, or Dunn's). The two-way ANOVA test and the Bonferroni post hoc test were also applied for proliferation analysis. According to the data distribution, correlation analyses were then performed using Pearson's or Spearman's correlation coefficient (r). OS was defined as the interval from the date of diagnosis. PFS was defined as the interval from the date of diagnosis until the first day of disease progression. Kaplan-Meier method and multivariable Cox proportional-hazards regression analyses were performed to determine the association of RNF123, SerpinE1, or concurrent RNF123 and SerpinE1 expression with overall survival. Cumulative incidence functions (CIF) and multivariable competing risk regression analyses were performed to evaluate the association of RNF123, SerpinE1, or concurrent RNF123 and SerpinE1 expression with recurrence-free survival, taking the competing risk of death into account, using statistical R package "cmprsk" [4,5]. The risk factors including age, gender, MGMT methylation status, and KPS were evaluated in the univariate and multivariable survival analyses [6]. A two-sided *p*-value < 0.05 was considered statistically significant: * $p < 0.05$; ** $p < 0.01$; *** $p < 0.001$ and NS = non-significant. All statistical analyses were performed with GraphPad Prism 5 (GraphPad software Inc.,

La Jolla, CA, USA) or R version 3.5.0 (R Core Team, 2018). The figures were made using CorelDraw graphics suite 8X.

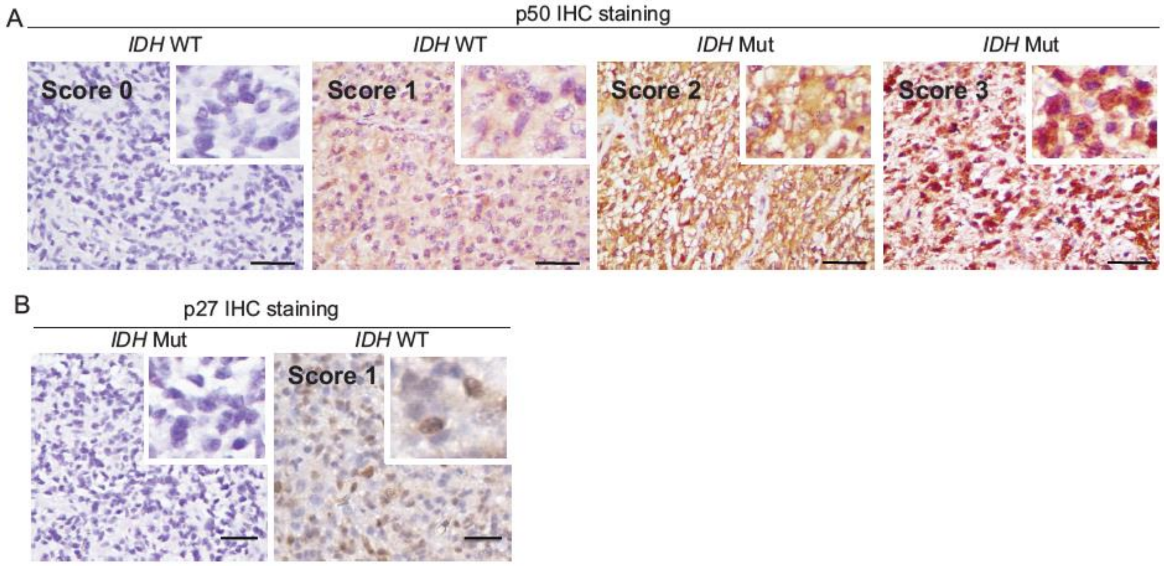

Figure S1. Immunohistochemistry analysis of p50 and p27 in GB**.** (**A**) Representative images of IHC showing the scores (0, 1, 2, or 3) for p50 staining in FFPE samples of our GB patient cohort. Scale bar = 50 µm. (**B**) Representative images of IHC showing the scores (0, or 1) for p27 staining in FFPE samples. Scale bar = 50 µm.

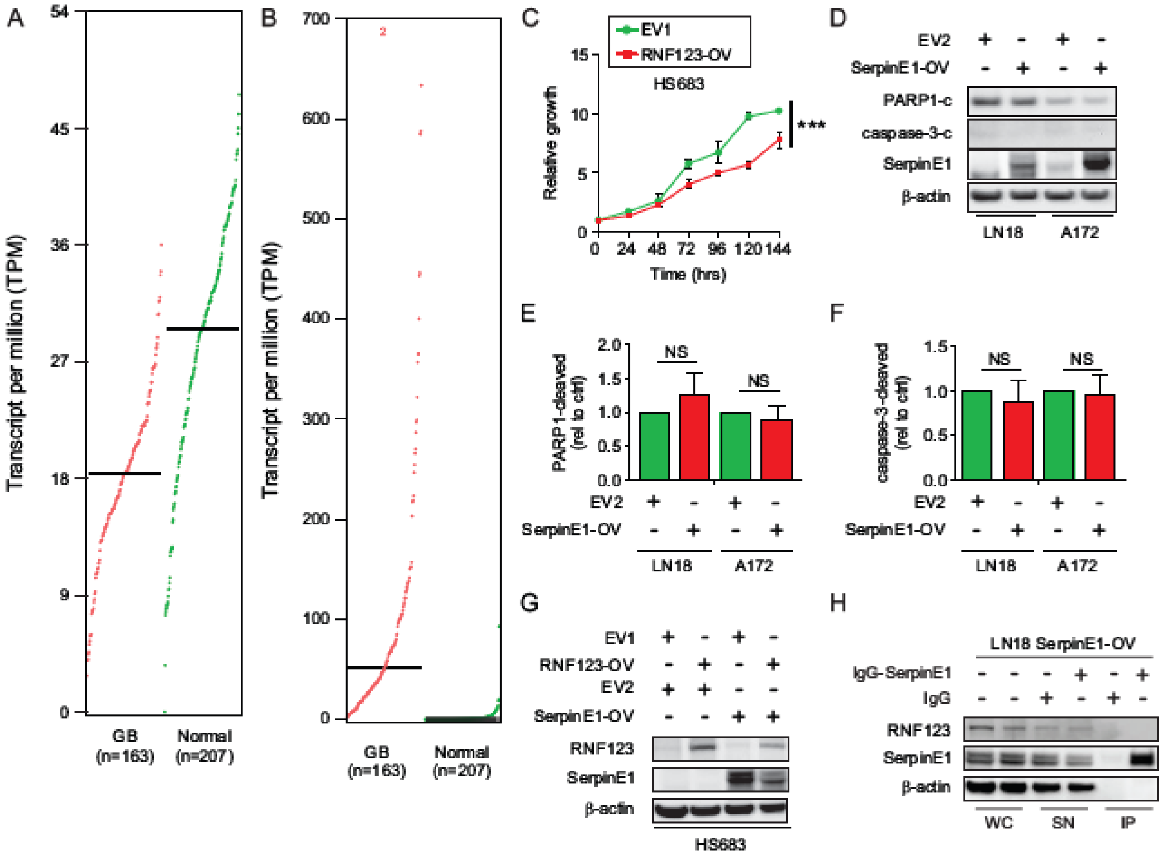

Figure S2. SerpinE1 and RNF123 expression in GB. Role of SerpinE1 in modulating apoptosis and controlling RNF123 expression. (A-B) RNF123 (A) and SerpinE1 (B) expression in GB (TCGA, $n = 163$) and normal brain tissue (GTEX, $n = 207$) using GEPIA database. (C) Proliferation of HS683 cell lines

with RNF123-OV or the empty vector (EV1) (two-way ANOVA, Bonferroni correction *** $p < 0.001$). (D) LN18 and A172 cell lines were stably overexpressing SerpinE1 (SerpinE1-OV) or the empty vector (EV2). PARP1 cleaved, caspase-3 cleaved, and SerpinE1 were assessed by western blot. β-actin was used as a loading control. (E-F) Quantification of PARP1 cleaved (E) or caspase-3 cleaved (F) in LN18 or A172 cell lines with SerpinE1 overexpression (SerpinE1-OV) or the empty vector (EV2) (*t*-test, NS= non-significant). (G) HS683 cell lines were stably transfected with constructs expressing EV1 + EV2, RNF123-OV + EV2, EV1 + SerpinE1-OV, or RNF123-OV and SerpinE1-OV. SerpinE1 and RNF123 expression levels were determined by western blot, and β-actin was used as a loading control. (H) Immunoprecipitation (IP) assay for SerpinE1 in LN18 cell lines. RNF123 and SerpinE1 were detected by western blot in the whole-cell (WC), supernatant after IP (SN) and IP fraction. β-actin was used as the loading control. Error bars represent mean ± SD from replicates ($n = 3$).

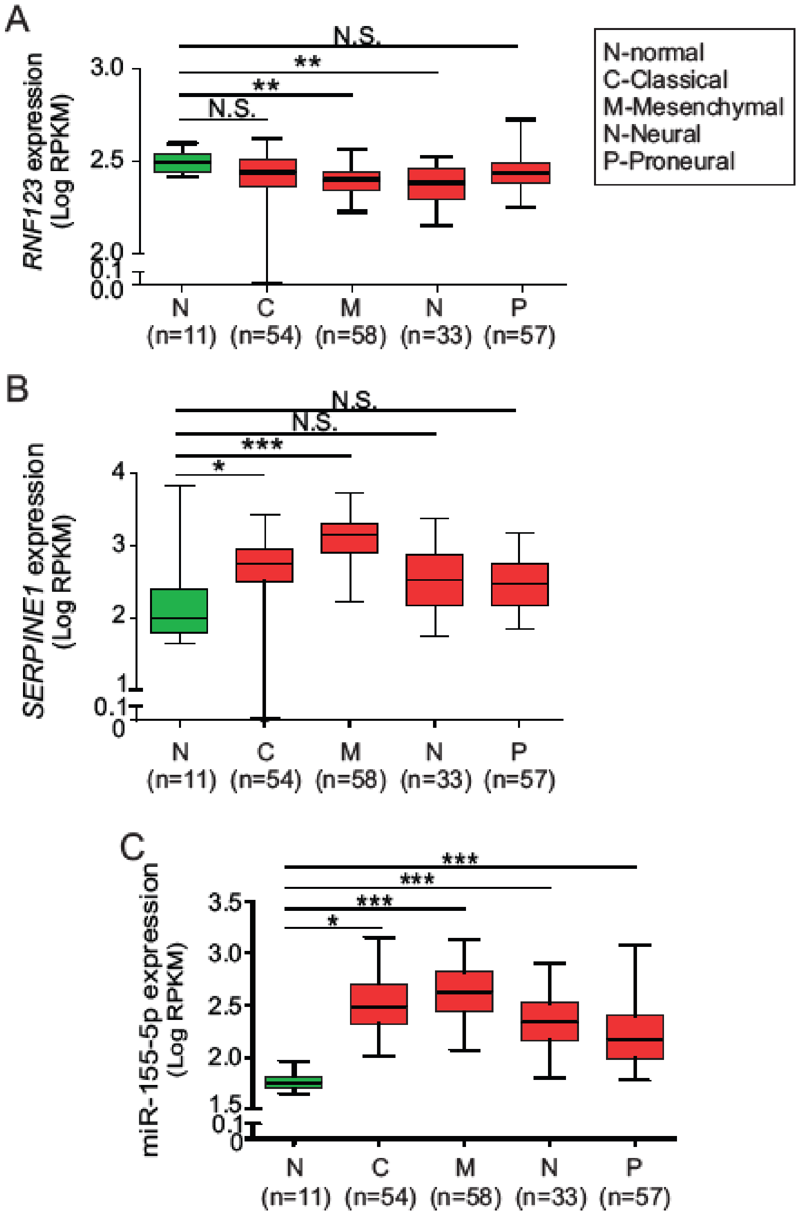

Figure S3. GB with mesenchymal subtype showed increased expression of *SERPINE1*. (A–C) TCGA dataset of GB patients was split according to their subtypes (normal (N, $n = 11$); classical (C, $n = 54$), mesenchymal (M, $n = 58$); neural (N, $n = 33$); proneural (P, $n = 57$)) based on RNA-sequencing and compared for *RNF123* (**A**), *SERPINE1* (**B**), and miR-155-5p (**C**) expression (Kruskal-Wallis test, Dunn's post hoc test * $p < 0.05$, ** $p < 0.01$, *** $p < 0.001$, NS = non-significant).

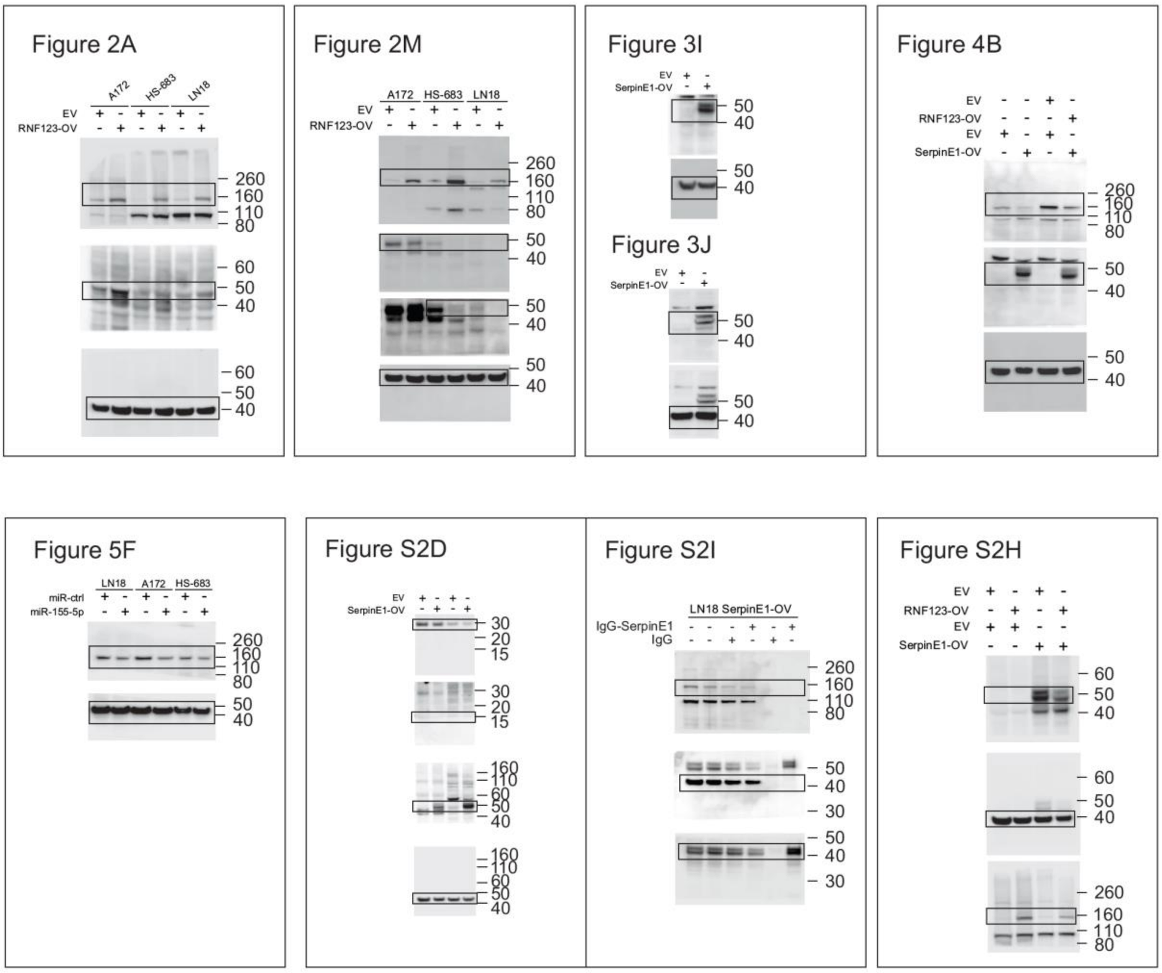

Figure S4. Western blot uncropped images used in the manuscript**.** Summary of all the uncropped images used in the manuscript. The Figure number where the images were utilized is indicated for each image.

Table S1A. Clinical pathological features of GB patients from TMA1.

Variables	*n* (%)
**RNF123 (H score)**	
High (<35)	50 (50)
Low (≥35)	50 (50)
**Age, year, mean (sd)**	56.19 (14.03)
**Gender**	
Male	68 (68)
Female	32 (32)
IDH	
WT	92 (92)
Mutated	8 (8)
***MGMT* methylation**	
Negative	40 (40)
Positive	60 (60)
**KPS**	
>85	84 (84)
<85	16 (16)

Table S1B. Univariate and Multivariable analysis for the association between RNF123 expression and overall survival.

Variables	Univariate				Multivariable			
	***HR**	**Lower 95% CI**	**Upper 95% CI**	***p*-value**	***HR**	Lower 95% CI	Upper 95% CI	*p*-value
**RNF123 (H score), median cutoff**								
High (≥35)	1				1			
Low (<35)	2.20	1.36	3.55	*0.001*	1.86	1.14	3.05	*0.01*
**Age** (year)	1.01	1	1.03	0.17	1.01	0.99	1.03	0.19
**Gender**								
Male	1				1			
Female	0.75	0.47	1.22	0.25	0.85	0.51	1.41	0.53
**KPS**								
>85	1				1			
<85	2.18	1.24	3.83	*0.01*	2.15	1.15	4.02	*0.02*
***MGMT* methylation**								
Negative	1				1			
Positive	0.98	0.63	1.53	0.92	1.17	0.74	1.85	0.51

* Hazard ratio form Cox proportional-regression analysis, CI = confidence interval.

Table S2A. Univariate and Multivariable Analysis for the Association between RNF123 expression and Recurrence-free Survival using Competing Risk Regression.

Variables	Univariate				Multivariable			
	**SHR***	**Lower 95% CI**	**Upper 95% CI**	***p*-value**	**SHR***	Lower 95% CI	Upper 95% CI	*p*-value
**RNF123 (H score), median cutoff**								
High (≥35)	1				1			
Low (<35)	2.36	1.54	3.61	*0.001*	2.16	1.41	3.33	*0.001*
**Age** (year)	1.01	1	1.03	0.12	1.01	0.99	1.02	0.31
**Gender**								
Male	1				1			
Female	0.67	0.43	1.04	0.08	0.72	0.48	1.10	0.13
**KPS**								
>85	1				1			
<85	1.12	0.61	2.04	0.72	1.16	0.59	2.30	0.67
***MGMT* methylation**								
Negative	1				1			
Positive	1.29	0.86	1.94	0.22	1.36	0.93	1.99	0.11

* Subdistribution hazard ratio from competing risk regression analysis, CI = confidence interval.

Table S2B. Clinical pathological features of GB patients from TMA2.

**Variables**	***n* (%)**
**SerpinE1 (H score)**	
Low (<145)	50 (50)
High (≥145)	50 (50)
**Age, year, mean (sd)**	56.19 (14.18)
**Gender**	
Male	72 (72)
Female	28 (28)
IDH1	
WT	91 (92)
MT	9 (8)
**KPS**	
>85	84 (84)
<85	16 (16)
***MGMT* methylation**	
Negative	38 (38)
Positive	62 (62)

Table S3A. Univariate and Multivariable Analysis for the Association Between SerpinE1 expression and Overall Survival.

**Variables**	**Univariate**				**Multivariable**			
	***HR**	**Lower 95% CI**	**Upper 95% CI**	***p*-value**	***HR**	Lower 95% CI	Upper 95% CI	*p*-value
**SerpinE1 (H score), median cutoff**								
Low (<145)	1				1			
High (≥145)	2.3	1.47	3.6	*<0.001*	2.41	1.48	3.92	*<0.001*
**Age** (year)	1.01	0.99	1.02	0.30	1.01	0.99	1.03	0.12
**Gender**								
Male	1				1			
Female	0.62	0.37	1.02	0.06	0.76	0.46	1.30	0.33
**KPS**								
>85	1				1			
<85	2.32	1.27	4.23	*0.01*	2.26	1.21	1.30	0.33
**MGMT methylation**								
Negative	1				1			
Positive	1.13	0.72	1.78	0.60	1.86	1.12	3.10	*0.02*

* Hazard ratio from Cox proportional-regression analysis, CI= confidence interval.

Table S3B. Univariate and Multivariable Analysis for the Association between SerpinE1 expression and Recurrence-free Survival using Competing Risk Regression.

**Variables**	**Univariate**				**Multivariable**			
	**SHR***	**Lower 95% CI**	**Upper 95% CI**	***p*-value**	**SHR***	Lower 95% CI	Upper 95% CI	*p*-value
**SerpinE1 (H score), median cutoff**								
Low (<145)	1				1			
High (≥145)	1.62	1.08	2.43	*0.02*	1.86	1.18	2.95	*0.01*
**Age** (year)	1.01	0.99	1.02	0.25	1.01	1	1.03	0.07
**Gender**								
Male	1				1			
Female	0.7	0.45	1.09	0.11	0.85	0.54	1.35	0.50
**KPS**								
Male	1				1			
Female	1.39	0.74	2.63	0.30	1.33	0.68	2.61	0.41
***MGMT* methylation**								
Negative	1				1			
Positive	1.31	0.87	1.98	0.19	1.91	1.17	3.11	*0.01*

* Subdistribution hazard ratio from competing risk regression analysis, CI= confidence interval.

Table S4A. Clinical pathological features of GB patients from TMA1 and TMA2.

**Variables**	***n*** (%)
**RNF123 (H score)**	
Low (<35)	25 (29.41)
High (≥35)	19 (22.35)
Others	41 (48.24)
**Age, year, mean (sd)**	56 (14.64)
**Gender**	
Male	61 (71.76)
Female	24 (24.24)
IDH	
WT	79 (92.94)
Mutated	6 (7.06)
***MGMT* methylation**	
Negative	34 (40)
Positive	51 (60)
**KPS**	
>85	73 (85.88)
<85	12 (14.12)

Table S4B. Univariate and Multivariable Analysis for the Association Between combined RNF123/SerpinE1 expression and Overall Survival.

Variables	Univariate				Multivariable			
	HR*	Lower 95% CI	Upper 95% CI	*p*-value	HR*	Lower 95% CI	Upper 95% CI	*p*-value
**RNF123/SerpinE1 (H score)**								
High RNF123 / Low SerpinE1	1				1			
Low RNF123 / High SerpinE1	2.69	1.65	4.39	*<0.001*	2.93	1.7	5.05	<0.001
Other	2.08	1.19	3.65	0.*01*	1.99	1.13	3.51	0.02
**Age** (year)	1.01	1	1.03	0.09	1.01	1	1.03	0.17
**Gender**								
Male	1				1			
Female	0.72	0.44	1.17	0.18	0.89	0.56	1.42	0.63
**KPS**								
>85	1				1			
<85	1.41	0.71	2.79	0.32	1.42	0.66	3.05	0.37
***MGMT* methylation**								
Negative	1				1			
Positive	1.23	0.8	1.9	0.35	1.79	1.09	2.92	0.02

Table S5. Univariate and Multivariable Analysis for the Association between combined RNF123/SerpinE1 expression and Recurrence-free Survival using Competing Risk Regression.

Variables	Univariate				Multivariable			
	SHR*	Lower 95% CI	Upper 95% CI	*p*-value	SHR*	Lower 95% CI	Upper 95% CI	*p*-value
**RNF123/SERPINE1 (H score)**								
High RNF123 / Low SerpinE1	1				1			
Low RNF123 / High SerpinE1	3.79	1.85	7.76	*<0.001*	3.56	1.61	7.83	*<0.001*
**Age** (year)	1.01	1	1.03	0.11	1.01	0.99	1.03	0.33
**Gender**								
Male	1				1			
Female	0.7	0.41	1.2	0.19	0.89	0.5	1.57	0.69
**KPS**								
>85	1				1			
<85	2.35	1.25	4.4	*0.01*	2.04	1.04	4.02	*0.04*
***MGMT* methylation**								
Negative	1				1			
Positive	0.96	0.59	1.55	0.86	1.45	0.84	2.5	0.19

* Subdistribution hazard ratio from competing risk regression analysis, CI = confidence interval.

Table S6. Univariate and Multivariable Analysis for the Association Between RNF123 and SerpinE1 expression with Overall Survival.

Variables	Univariate				Multivariable			
	HR*	Lower 95% CI	Upper 95% CI	*p*-value	HR*	Lower 95% CI	Upper 95% CI	*p*-value
**RNF123 (H-score)**								
High RNF123 (≥35)	1				1			
Low RNF123 (<35)	2.12	1.26	3.55	*0.004*	1.59	0.93	2.72	0.09
**SerpinE1 (H score)**								
High RNF123 (≥145)	1				1			
Low RNF123 (<145)	2.03	1.26	3.28	*0.004*	2.01	1.18	3.44	0.01
**Age** (year)	1.01	1	1.03	0.11	1.01	0.99	1.03	0.19
**Gender**								
Male	1				1			
Female	0.7	0.41	1.2	0.19	0.84	0.48	1.48	0.55
**KPS**								

>85	1				1			
<85	2.35	1.25	4.4	0.19	2.27	1.14	4.50	*0.02*
***MGMT* methylation**								
Negative	1				1			
Positive	0.96	0.59	1.55	0.86	1.63	0.93	2.84	0.09

* Hazard ratio from Cox proportional-regression analysis, CI = confidence interval.

References

1. Bustos, M.A.; Ono, S.; Marzese, D.M.; Oyama, T.; Iida, Y.; Cheung, G.; Nelson, N.; Hsu, S.C.; Yu, Q.; Hoon, D.S.B. MiR-200a Regulates CDK4/6 Inhibitor Effect by Targeting CDK6 in Metastatic Melanoma. *J. Invest. Dermatol.* **2017**, 10.1016/j.jid.2017.03.039, doi:10.1016/j.jid.2017.03.039.
2. Iida, Y.; Ciechanover, A.; Marzese, D.M.; Hata, K.; Bustos, M.; Ono, S.; Wang, J.; Salomon, M.P.; Tran, K.; Lam, S., et al. Epigenetic Regulation of KPC1 Ubiquitin Ligase Affects the NF-kappaB Pathway in Melanoma. *Clin. Cancer Res.* **2017**, *23*, 4831–4842, doi:10.1158/1078-0432.ccr-17-0146.
3. Love, M.I.; Huber, W.; Anders, S. Moderated estimation of fold change and dispersion for RNA-seq data with DESeq2. *Genome Biol.* **2014**, *15*, 550, doi:10.1186/s13059-014-0550-8.
4. Scrucca, L.; Santucci, A.; Aversa, F. Regression modeling of competing risk using R: an in depth guide for clinicians. *Bone Marrow Transplant.***2010**, *45*, 1388–1395, doi:10.1038/bmt.2009.359.
5. Scheike, T.H.; Zhang, M.J. Analyzing Competing Risk Data Using the R timereg Package. *J. Stat. Softw.* **2011**, *38*.
6. Hidalgo, B.; Goodman, M. Multivariate or multivariable regression? *Am. J. Public Health* **2013**, *103*, 39–40, doi:10.2105/ajph.2012.300897.

© 2020 by the authors. Licensee MDPI, Basel, Switzerland. This article is an open access article distributed under the terms and conditions of the Creative Commons Attribution (CC BY) license (http://creativecommons.org/licenses/by/4.0/).
